# Leave no stone unturned: individually adapted xerotolerant *Thaumarchaeota* sheltered below the boulders of the Atacama Desert hyperarid core

**DOI:** 10.1186/s40168-021-01177-9

**Published:** 2021-11-26

**Authors:** Yunha Hwang, Dirk Schulze-Makuch, Felix L. Arens, Johan S. Saenz, Panagiotis S. Adam, Christof Sager, Till L. V. Bornemann, Weishu Zhao, Ying Zhang, Alessandro Airo, Michael Schloter, Alexander J. Probst

**Affiliations:** 1 Astrobiology Group, Center for Astronomy & Astrophysics, Technische Universität Berlin, 10623 Berlin, Germany; 2 Environmental Microbiology and Biotechnology, Department of Chemistry, University of Duisburg-Essen, 45141 Essen, Germany; 3 Department of Organismic and Evolutionary Biology, Harvard University, Cambridge, MA USA; 4 Section Geomicrobiology, German Research Centre for Geosciences (GFZ), 14473 Potsdam, Germany; 5 Department of Experimental Limnology, Leibniz-Institute of Freshwater Ecology and Inland Fisheries (IGB), 12587 Stechlin, Germany; 6 School of the Environment, Washington State University, Pullman, WA 99164 USA; 7 Research Unit for Comparative Microbiome Analysis, Helmholtz Zentrum München, 85758 Oberschleißheim, Germany; 8 Department of Cell and Molecular Biology, College of the Environment and Life Sciences, University of Rhode Island, Kingston, RI USA; 9 Centre of Water and Environmental Research (ZWU), University of Duisburg-Essen, Universitätsstraße 5, 45141 Essen, Germany

**Keywords:** Atacama, Hyperaridity, Archaea, Soil microbiome, Xerotolerance

## Abstract

**Background:**

The hyperarid core of the Atacama Desert is an extremely harsh environment thought to be colonized by only a few heterotrophic bacterial species. Current concepts for understanding this extreme ecosystem are mainly based on the diversity of these few species, yet a substantial area of the Atacama Desert hyperarid topsoil is covered by expansive boulder accumulations, whose underlying microbiomes have not been investigated so far. With the hypothesis that these sheltered soils harbor uniquely adapted microbiomes, we compared metagenomes and geochemistry between soils below and beside boulders across three distantly located boulder accumulations in the Atacama Desert hyperarid core.

**Results:**

Genome-resolved metagenomics of eleven samples revealed substantially different microbial communities in soils below and beside boulders, despite the presence of shared species. Archaea were found in significantly higher relative abundance below the boulders across all samples within distances of up to 205 km. These key taxa belong to a novel genus of ammonia-oxidizing *Thaumarchaeota*, *Candidatus* Nitrosodeserticola. We resolved eight mid-to-high quality genomes of this genus and used comparative genomics to analyze its pangenome and site-specific adaptations. *Ca.* Nitrosodeserticola genomes contain genes for ammonia oxidation, the 3-hydroxypropionate/4-hydroxybutyrate carbon fixation pathway, and acetate utilization indicating a chemolithoautotrophic and mixotrophic lifestyle. They also possess the capacity for tolerating extreme environmental conditions as highlighted by the presence of genes against oxidative stress and DNA damage. Site-specific adaptations of the genomes included the presence of additional genes for heavy metal transporters, multiple types of ATP synthases, and divergent genes for aquaporins.

**Conclusion:**

We provide the first genomic characterization of hyperarid soil microbiomes below the boulders in the Atacama Desert, and report abundant and highly adapted *Thaumarchaeaota* with ammonia oxidation and carbon fixation potential. *Ca.* Nitrosodeserticola genomes provide the first metabolic and physiological insight into a thaumarchaeal lineage found in globally distributed terrestrial habitats characterized by various environmental stresses. We consequently expand not only the known genetic repertoire of *Thaumarchaeota* but also the diversity and microbiome functioning in hyperarid ecosystems.

Video Abstract

**Supplementary Information:**

The online version contains supplementary material available at 10.1186/s40168-021-01177-9.

## Background

Deserts are widespread terrestrial ecosystems, where aridity limits the proliferation and diversification of life [1]. Despite the paucity of macrofauna in desert ecosystems, previous studies (e.g., on the Sahara Desert [2, 3], the Namib Desert [4]**,** and the Antarctic Dry Valley [5–7]) detected highly adapted microbial communities, suggesting that the major bioprocesses in deserts are driven by microbial life [8]. The Atacama Desert is the oldest nonpolar desert on Earth [9] and its long history of hyperaridity has resulted in the accumulation of atmospheric salts in its soils [10]. In particular, the surface soils in the hyperarid core [11] of the Atacama Desert are characterized by extreme desiccation (water content < 1% by weight), low water activity as a consequence of high salt content, and high UV irradiation (~ 30 J·m^−2^) [12]. Low yields of DNA have been isolated and analyzed in previous studies [12–15] revealing sparse microbial communities with low diversity, dominated by *Actinobacteria* and *Firmicutes* [10]. While recent studies showed that some of these microbes are viable and actively replicating, as indicated by cultivation experiments [13] and in situ replication measures (iRep [16];) [12] respectively, very little is known about the carbon and nitrogen cycling in the hyperarid soils of the Atacama Desert. To date, only localized carbon fixation could be inferred from the findings of hypolithic and endolithic cyanobacteria [17, 18], but no information on possible pathways for the transformation of other nutrients has been obtained so far.

*Thaumarchaeota* mediate important environmental processes in both marine and terrestrial ecosystems and are particularly adapted to oligotrophic environments with their highly energy-efficient carbon fixation pathway [19, 20]. *Thaumarchaeota* have been detected in hot desert soils (e.g., Mojave Desert, California and Chihuahuan Desert, New Mexico) [21]. However, previous studies reported the general pattern of decreasing archaeal diversity with increasing aridity [22, 23] and most in-depth desert microbiome surveys focused on bacterial communities [24, 25]. In particular, microbiome studies of hyperarid deserts in Antarctica reported the absence [24, 26] or low abundance [27] of Archaea suggesting lower tolerance of Archaea to hyperaridity. The Atacama Desert soil microbiome has previously been thought to be dominated by Bacteria, with an exception of halophilic Archaea (*Halobacteriales*) in locations such as coastal soils [12] and salt crusts [28]. Thaumarchaeal 16S rRNA gene sequences have been detected in the Atacama region, such as in playas and alluvial fans after a heavy rainfall [29], and in high-elevation mineral soils of Volcán Llullaillaco [30]. However, the metabolic potential, adaptations and the abundance (relative to Bacteria) of these *Thaumarchaeota* could not be resolved in the aforementioned studies.

The Atacama Desert hyperarid core harbors many expansive boulder fields [31–33] where individual boulders are up to 37 millions of years old [34, 35]. Despite their ubiquity and the uniquely sheltered conditions beneath the boulders in the Atacama Desert, no study has determined the microbial and geochemical compositions of soils below the boulders. We compared the metagenomes and geochemistry of soils below and beside the boulders, with the hypothesis that extended periods of physical shelter and isolation by the boulder cover would result in distinct ecosystems that support a unique composition of organisms protected from harsh environmental stressors (i.e., radiation and resulting reactive oxygen species [ROS], temperature and humidity fluctuations, deposition of salts). Community structure and metabolic potentials inferred from genome-resolved metagenomics were interpreted in conjunction with geochemical measurements in order to characterize and compare the ecological significance of microbiomes found in the two sample types. *Thaumarchaeota* were one of the key taxa differentiating microbial communities inhabiting below and beside the boulders. Consequently, thaumarchaeal genomes were selected for an in-depth pangenomic analysis, revealing their potential for carbon and nitrogen cycling. We further compared them to their closest known relatives and to each other, to unveil possible adaptations to these uniquely protected, sparsely populated, and constantly selective environments.

## Results and discussion

### Hyperarid soils sheltered under the boulders are geochemically distinct and organic carbon deficient

**Fig. 1:**
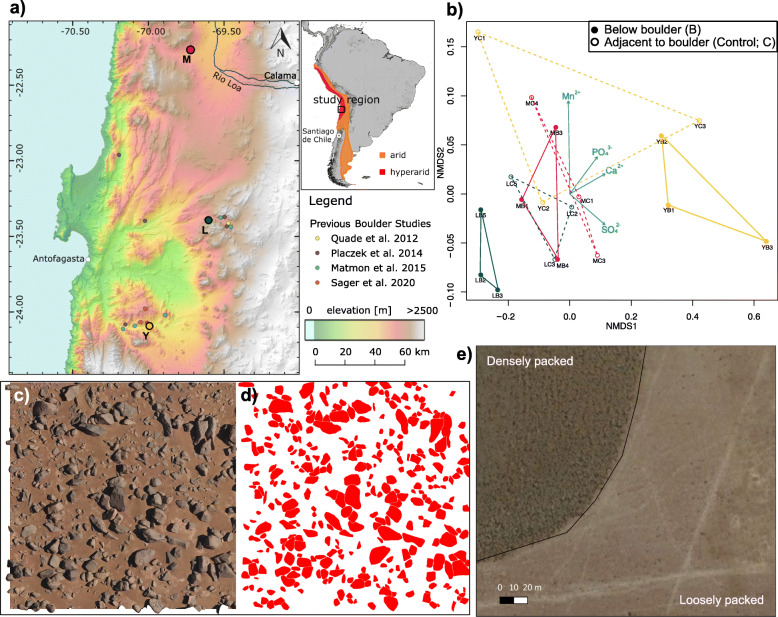
Sampling location and soil geochemistry. **a** Location of three sampling sites and their abbreviations in parentheses. Locations of previously studied boulder fields are mapped and their references are shown in the legend. Top-right corner maps the arid and hyperarid climate regions across South America [11]. **b** Non-metric multidimensional scaling (NMDS) ordination (stress = 0.024) of anion and cation concentrations in each soil sample. Different colors represent different sampling sites (green = L, red = M, yellow = Y). Filled vs unfilled data points correspond to the sample type information (below (B) or beside boulder (C)). Blue vectors represent fitted ion species onto the ordination with adjusted *p*-value < 0.05. **c** An example drone image used to map individual boulders at high resolution; raw data collected by Sager et al. [33] and reanalyzed in this study. Image frame corresponds to 15 x 15 m. **d** Corresponding mapping of individual boulders imaged in c, same scale. **e** Satellite image distinguishing “densely” and “loosely” packed boulder accumulations

Boulder accumulations in the Atacama Desert are both frequent and expansive (see Supplementary Results R1 and Figures S1-3 for estimates of boulder coverage at different resolutions). Previous geomorphological studies [31–33] (Fig. 1a) have hypothesized a seismic origin of these boulder accumulations typically found at the base of hills and valley floors, both of which are abundant landforms in the hyperarid core (Figure S3) [36]. We used satellite images to map the presence of boulder accumulations in regions within 5 km to our sampling sites and estimated between 16 and 31% in each of the studied regions to exhibit boulder accumulations of various densities (Figure S2). For instance, in densely packed regions the boulders covered up to 21% of the topsoil (Fig. 1c–e). Soils below the boulders are subject to lower diurnal temperatures and lower fluctuations in relative humidity than those directly beside the boulders (Figure S4a-c). Based on the dew point temperature calculations, we showed that the condensation in the morning is far less likely for soil below boulders compared to soil beside boulders (Figure S4d-f), suggesting that water content below boulders may be even lower than in previously studied Atacama Desert hyperarid top soils (~ 0.2% by weight) [12].

We compared soil samples of two sample types: soils taken below boulders (B) and soils taken beside the boulders (control, C) at three different sampling locations (Lomas Bayas: L, Maria Elena: M, Yungay Valley Y) (Fig. 1a, Table S1). While the collected soils were mineralogically very similar with some variation per sampling location (Figure S5), their ion concentrations showed large variance between boulder fields, individual boulders, and sample types. Interestingly, B samples clustered based on their sampling location along the NMDS1 axis, while C samples showed little pattern based on their sampling sites (Fig. 1b). In general, samples from locations L and M were enriched in F^−^, while Y samples were enriched in PO_4_^3−^, SO_4_^2−^, Mn^2+^ and Ca^2+^, suggesting boulder field specific patterns of ion concentrations. More sampling location dependent ion composition patterns among the B samples indicate that soils below the boulder are sheltered from external input of ions (i.e., atmospheric deposition of salts), thereby exhibiting a more representative ion composition patterns of the underlying soils in the sampling site. When comparing the B and C sample of each individual boulder, nitrate ion concentrations were significantly lower in B samples compared to C samples (paired Welch’s *t*-test; NO_3_^−^: *t*(8) = − 3.9, adjusted *p*-value = 0.027, Figure S6). Total Organic Carbon (TOC) concentrations were at or below detectable levels in both below boulder and beside boulder samples (Figure S7). Our results show that the soils below the boulders are not only hyperarid and organic carbon deficient, but also sheltered from the atmospheric input of both water (e.g., fog, dew) and salts.

### The microbial community in the Atacama Desert hyperarid core shows evidence of in situ replication

We conducted genome-resolved metagenomics on the eleven successfully prepared metagenomes (for details, see Material and Methods and Table S2): three B and three C samples came from Lomas Bayas (LB2, LB3, LB5 and LC2, LC3, LC5), three B samples were from Maria Elena (MB1, MB3, MB4) and two B samples from Yungay Valley (YB1, YB3). Across these eleven samples, we binned 67 medium to high quality (> 75% completeness, < 10% contamination) metagenome-assembled genomes (MAGs). Eight of these genomes were thaumarchaeal with completeness ranging from 84.67 to 98.54%. The other 59 mid-to-high quality MAGs belonged to *Actinobacteria* (*n* = 28), *Chloroflexi* (*n* = 29), *Firmicutes* (*n* = 1), and *Alphaproteobacteria* (*n* = 1) (see concatenated protein trees of mid-to-high bacterial MAGs in Figure S8 and archaeal MAGs in Fig. 4). In situ replication measures [iRep [16]] and GRiD [37] were successfully calculated for 30 and 36 out of all mid-to-high-quality bacterial genomes (*n* = 59), respectively, indicating an active metabolism of the majority of the indexed population (filtered iRep values ranged between 1.34 and 3.47, median: 1.89; refined GRiD values ranged between 1.13 and 3.12, median: 2.07; for comparison, see Figure S9). On average, genomes recovered from below boulder metagenomes were associated with slightly higher iRep and GRiD values than the control metagenomes of the same site (p-value < 0.05, Welch’s t-test for LB and LC MAGs**)**. A full overview of genome statistics, their taxonomic classification and corresponding iRep and GRiD values is provided in Table S3.

### Atacama soils below boulders harbor unique microbial communities with a high relative abundance of Thaumarchaeota

We detected 147 different bacteria and Archaea based on clustering of S3 ribosomal proteins (RpS3, 99% identity, Table S4, Figure S10). Species evenness and alpha diversity were similar across samples with no statistically significant differentiation between sites or sample types (Figure S11). However, principal coordinate analysis (PCoA) of the communities (Figure S12a) demonstrated clustering of samples based on sample site (L, M, Y) as well as sample type (B, C). This was corroborated by the Multiple Response Permutation Procedure (MRPP) indicating significant influence on the community structure by both sampling location (chance corrected within group agreement *A* = 0.2648, significance of delta = 0.001) and sample type (*A* = 0.1488, significance of delta = 0.002). Using BioENV [38], we identified F^−^ concentration to be most correlated (Spearman’s rho = 0.582) with the community composition. F^−^ has been shown to be correlated with microbial community structures in previous Atacama soil studies [29], as well as in other environments such as groundwater [39]. Despite the low concentrations of F^−^ (~5 mg/g soil for values above the detection limit) across soils, we identified on average 15 (± 3) putative fluoride transporters CrcB per metagenome suggesting that fluoride detoxification may play a role in shaping the Atacama Desert microbial community. Additionally, we conducted a NMDS analysis (Figure S12b), identifying additional ions (Ca^2+^, SO_4_^2−^) that could be correlated with the community composition (adjusted *p*-value < 0.05).

**Fig. 2:**
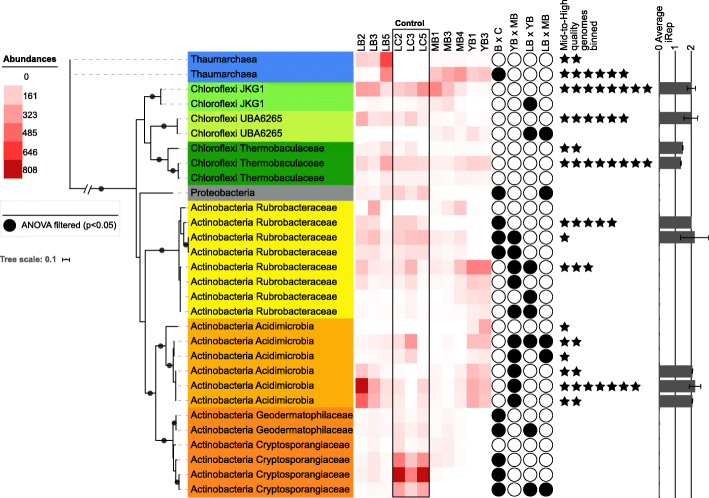
Phylogenetic tree of 30 most abundant taxa (*rpS3* clusters) out of 147 and their normalized abundances across all samples. Filled stars represent the number of *rpS3* sequences in the cluster that were successfully binned in mid-to-high quality genomes and gray bars indicate the average iRep values calculated for the mid-to-high quality genomes. Strongly supported branches as described in the “Materials and Methods” section are indicated with black dots

Thirty-three of the identified taxa differed significantly in their abundances (ANOVA [40], *p*-value < 0.05) between B and C samples. Such taxa included *Actinobacteria* (belonging to *Cryptosporangiaceae*, *Streptomycetaceae*, and *Geodermatophilaceae*), as well as one *Alphaproteobacteria* (*Acetobacteraceae*). These taxa were particularly abundant in C samples and nearly absent in B samples, suggesting specific and unknown selection processes for the two different sample types. Alternatively, some of these taxa may be deposited through aeolian transport [41]. Figure 2 shows the phylogenetic relationship among the top 30 most abundant taxa across the samples based on RpS3 proteins and links them to their respective MAGs as well as their differential coverage across the samples. We conclude that below boulder (B) and beside boulder (C) present substantially different habitats of the same ecosystem.

A thaumarchaeal OTU was the only taxon with higher abundance in below boulders samples than in control samples (ANOVA, *p*-value = 0.0396). All eight metagenomes constructed from samples retrieved below the boulders contained high abundances of *Thaumarchaeota*. Based on the ranked abundance of *rpS3* gene coverages (Figure S13), *Thaumarchaeota* ranked among the top seven most abundant taxa across all B samples. Based on *rpS3* gene coverages, the relative abundance of *Thaumarchaeota* varied between 4.5 and 34.6% across below boulders samples, with an average of 13.5%. In three samples (MB3, MB4, LB5), *Thaumarchaeota* were the most abundant organisms, e.g., in LB5, *Thaumarchaeota* were four-fold more abundant than the second most abundant taxon. The abundance of *Thaumarchaeota* under boulders and their near absence in control samples parallel the previous findings from marine environments, where thaumarchaeal abundance was observed to be anti-correlated with irradiation in the surface waters (e.g., increase in thaumarchaeal abundance in winter and decrease in summer, particularly in polar regions) [42–46]. To date, the underlying reason for the lower abundance of *Thaumarchaeota* in highly irradiated environments remains inconclusive. Three main hypotheses have been proposed for this phenomenon: (i) increased competition against phototrophs [44, 47], (ii) Photoinhibition of ammonia oxidation [48–50], and iii) indirect photoinhibition by reactive oxygen species (ROS) (i.e., hydrogen peroxide) [51]. When applying the first hypothesis to our study site, we posit that the near absence of *Thaumarchaeota* in the control samples is not due to the increased competition against phototrophs, based on the lack of phototrophs detected in the C samples. The second hypothesis is also not applicable for our study site as UV and photoradiation do not penetrate into the soil beyond the very surface of the topsoil, especially in the Atacama Desert where soils experience minimal perturbation. The third hypothesis, however, may be relevant to our study site; Although we did not directly measure ROS levels in our samples, photochemically produced ROS (H_2_O_2_ and metal superoxides and peroxides) have previously been found to accumulate in the Atacama Desert (Yungay site) top soils at levels an order of magnitude higher than in non-arid control soils [52]. Considering the limited half-life of ROS, we hypothesize that soils that have been covered under boulders for hundreds, if not thousands, of years harbor significantly lower concentrations of ROS. Further investigation correlating concentrations of specific ROS below and beside boulders with thaumarchaeal abundance (and activity) could explain the causal relationships between ROS levels [51] and thaumarchaeal abundance.

### Ammonia-oxidizing *Thaumarchaeota* occupy an important niche in the Atacama Desert carbon and nitrogen cycling

**Fig. 3:**
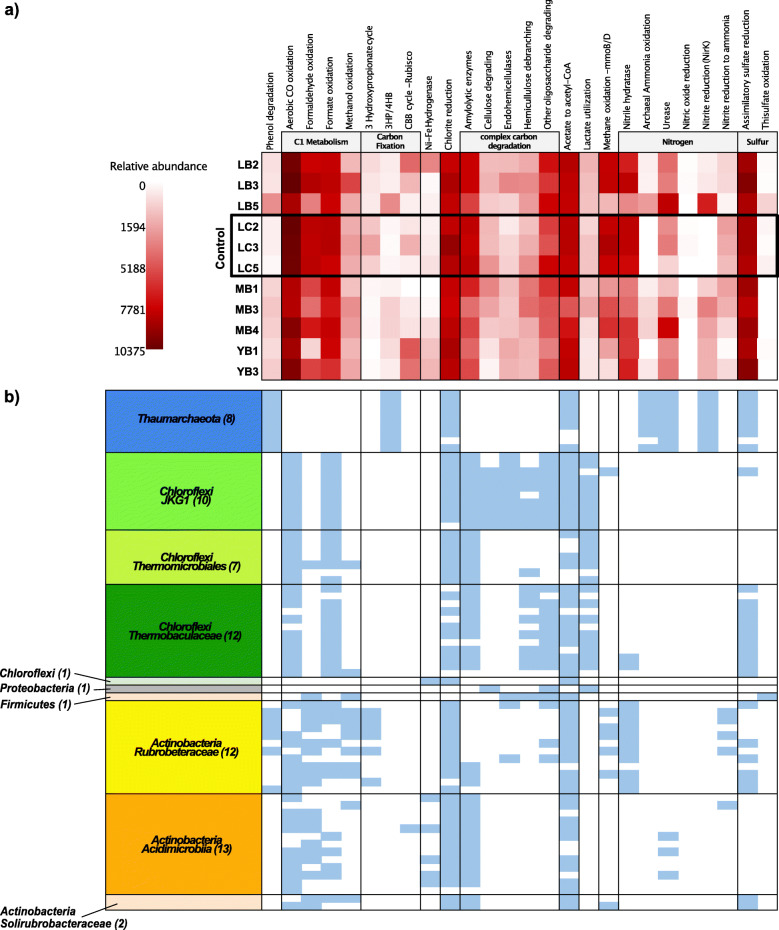
Metabolic potential prediction across samples and mid-to-high quality genomes. **a** Relative abundances of chemoautolithotrophic marker genes predicted using METABOLIC for each sample. **b** Presence (blue) and absence (white) of chemoautolithotropic marker genes in mid-to-high quality genomes. Genomes are clustered based on taxa and the number of genomes in each cluster is shown in parentheses in the row names

Relative abundances of key marker genes in the assembled metagenomes revealed the potential for carbon fixation, C1 metabolism, complex carbon degradation, and fermentation across all samples (Fig. 3). Notably, we detected eleven Form I RuBisCO sequences across ten out of eleven metagenomes. RuBisCO large subunits were mostly (nine out of eleven) situated next to a small subunit on very short contigs containing between one and five genes with an exception in the LC5 metagenome (Figure S14a). Regions that are difficult to assemble into longer contigs may exhibit differential kmer frequencies from the rest of the genome resulting from horizontal gene transfers, which has been observed to occur frequently for RuBisCO subunits [53, 54]. The RuBisCO large subunit sequences formed a novel subclade of type IA and were closely related to recently discovered RuBisCOs from the Negev desert [55] (Figure S14b). This phylogenetically distinct grouping of RuBisCO sequences from distantly located desert environments suggests that there exists a desert-specific ecotype of RuBisCO. We did not detect any key bacterial photosynthesis marker genes (e.g., *psaA*, *psbA*); instead, we detected 20 additional homologs of actinobacterial-type (group 1h) respiratory H_2_-uptake NiFe hydrogenases across all metagenomes. The presence and high abundance of RuBisCOs of subtype 1E and high-affinity Ni-Fe hydrogenase suggest that the hydrogen-driven CBB cycle may play a key role in carbon fixation in extreme conditions of the hyperarid desert soils where photosynthesis is inhibited. Our results complement recent findings by Bay et al. [55] that showed increasing H_2_ oxidation with increasing aridity in incubation experiments, providing evidence that hydrogenotrophic carbon fixation is more prevalent than photosynthesis in dry soils, similar to the novel chemosynthetic subclade IE RuBisCOs in antarctic soil [56] (Figure S14cd). In addition to the CBB pathway, we detected 3 hydroxypropionate cycle and 3HP/4HB pathway marker genes in our samples. The marker genes for the three carbon fixation pathways were differentially abundant between B and C samples. For instance, B samples contained higher relative abundances of 3HP/4HB pathway marker genes and lower relative abundances of 3-hydroxypropionate cycle marker genes than C samples (Kruskal-Wallis test, *p*-value = 0.012). Additionally, 3-hydroxypropionate cycle marker gene abundances were correlated with NO_3_^−^, Mg^2+^, and K^+^ ion concentrations (Pearson’s *R* = 0.75 − 0.78, adjusted *p*-value < 0.05).

Significant gaps in the potential for nitrogen cycling were observed. Archaeal ammonia oxidation potential was only found in below boulder samples (with the exception of YB1). Additionally, we found diverse sets of actinobacterial and chloroflexal ammonification proteins (Table S6). We found *norB* homologs in four out of eleven samples, but could not detect any *norC* in the same samples, possibly due to low abundance. No potential for nitrogen fixation, nitrate reduction as well as nitrite oxidation were identified in the metagenomes, indicating that these are not major processes contributing to the biological nitrogen cycling. The lack (or near absence) of nitrogen fixation and denitrification genes suggests low overall biological input and little loss of biologically available nitrogen. Although the investigated soils are known to be enriched in nitrates (particularly at ~ 1-m depth) that have accumulated over millions of years through abiotic processes (e.g., atmospheric formation through lightning followed by dry deposition and rainwater infiltration) [57], below boulder nitrate concentrations were significantly lower (Figure S6), likely due to the combined effect of microbial turnover and lack of atmospheric or hydrologic input.

Resolving the metabolic potential at the genomic level delineated the role each taxonomic group plays in these microbial communities characterized by low species diversity. The presence and absence of key metabolic genes for each mid-to-high quality genome are shown in Fig. 3b. Our analysis shows that although all samples show carbon fixation potential, taxa capable of fixing carbon are limited to *Thaumarchaeota* (through the 3HP/4HB pathway) and some *Actinobacteria* (3-hydroxypropionate pathway). Notably, one RuBisCO Form I (subtype 1A) could be binned to a high-quality *Acidomicrobiia* genome also containing a high-affinity H_2_-uptake Ni-Fe hydrogenase (group 1h). RuBisCO sequences detected in our study were also closely related to those binned in *Acidimicrobiia* MAGs (also encoding Ni-Fe hydrogenases) assembled from the Negev desert soil metagenomes (Figure S14b) [55], providing additional confirmation for the taxonomic assignment. Chloroflexi genomes associated with the lineage *JKG1* had the broadest potential of degrading complex carbon, suggesting a fermentative lifestyle, while *Actinobacteria* harbored genes involved in metabolizing a wide range of C1 substrates. Nitrite reduction potential detected in below boulder sites was constrained to the *nirK* genes found in *Thaumarchaeota*. NirK in *Thaumarchaeota* has been hypothesized to play a key role in ammonia oxidation [58, 59] and is biochemically capable of transforming N compounds to produce nitric oxide [60]. Furthermore, thaumarchaeal genomes indicated a potential for mixotrophy, featuring *ubiX* genes involved in phenol metabolism, as well as *acdA* and *acs* involved in acetate metabolism. It is important to note that while the presence of the key marker genes can be used to deduce the metabolic potential of these organisms, it cannot alone determine the functional physiology without further in situ measurements (i.e., gene and protein expression, stable isotope probing). In sum, our results indicate conserved metabolic capacities across genomes that belonged to the same taxonomic family, with thaumarchaeal genomes carrying unique metabolic pathways that could contribute to poorly understood nitrogen and carbon cycling in the Atacama hyperarid core.

### A novel genus of *Thaumarchaeota* with highly conserved core genome and diverse auxiliary genes

**Fig. 4:**
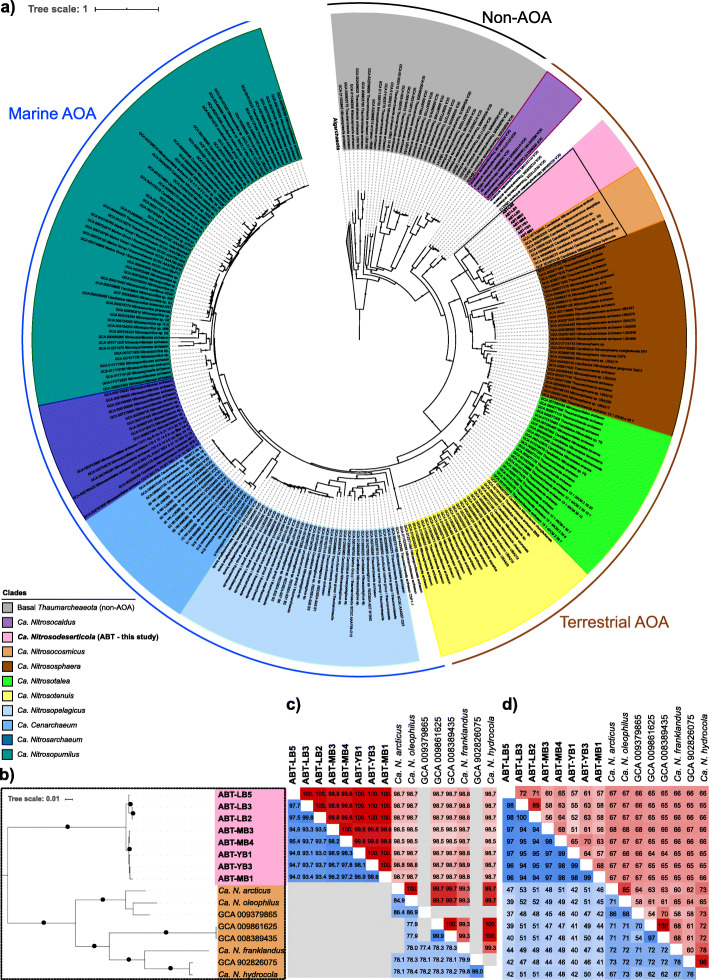
Phylogenomic placement of ABT genomes using 37 housekeeping single-copy genes. **a** Phylogenetic tree of 298 NCBI genomes annotated as *Thaumarchaeota* and eight ABT genomes. *Aigarchaeota* were identified and used as the outgroup. Black, brown and blue ranges distinguish whether organisms are ammonia-oxidizing Archaea (AOA) and their typical habitats (terrestrial vs marine). Strongly supported branches as described in the Materials and Methods section are indicated with black dots. **b** Magnified view of the branches placing the ABT (*Ca.* Nitrosodeserticola) genomes and its sister group *Ca.* Nitrosocosmicus*.* Strongly supported branches as described in the Materials and Methods section are indicated with black dots. **c** Lower-left (blue) triangle of the matrix corresponds to FastANI between genomes, where gray values indicate below calculation threshold (80% identity). Upper-right (red) triangle of the matrix corresponds to 16S rRNA gene identity values, where gray values are used for genomic bins without a 16S rRNA gene. **d** Lower-left (blue) triangle corresponds to the amino acid identity (AAI) and upper-right (red) triangle corresponds to the Orthologous Fraction (OF) between a pair of compared genomes

Eight mid-to-high quality Atacama Boulder Thaumarchaeal genomes (ABT) were assembled with an average GC content of 34.6% (± 0.1%) and average size of 2.5 Mbps (± 0.4 Mbps). Each genome contained on average 3,123 (± 579.7) predicted genes with a mean coding density of 71.8% (± 1.7%). The genomes were phylogenetically placed using 37 single-copy house-keeping genes, forming a monophyletic sister cluster to the recently characterized *Ca.* Nitrosocosmicus (Fig. 4a). The ABT clade and *Ca.* Nitrosocosmicus form a sister group to *Ca.* Nitrososphaera, a mesophilic terrestrial clade. Genomes from the same sites were more related to each other, with ABT-MB and ABT-YB genomes forming a separate branch from the ABT-LB genomes (Fig. 4b). Five ABT genomes contained a copy of the ammonia monooxygenase A (*amoA*) gene each (Table S6). On closer examination, two other genomes (ABT-MB3, ABT-MB4) contained conserved *amoA* regions, which failed in protein prediction due to ambiguous bases in scaffolding. No *amoA* sequences were found in ABT-YB1. Three additional unbinned *amoA* genes were detected across the metagenomes (MB3, MB4, YB3). Altogether, the eight *amoA* nucleotide sequences were 100% identical in their amino acid sequences to each other and to previously published *amoA* sequences from *Ca.* Nitrosocosmicus oleophilus and *Ca.* Nitrosocosmicus hydrocola, which had been phylogenetically identified to be one of the basal clades of archaeal *amoA* after *Ca.* Nitrosocaldus [61]. Figure S15a resolves the nucleotide level phylogenetic placement of binned *amoA* sequences as well as unbinned *amoA* sequences recovered from the sample metagenomes. Interestingly, one *amoA* recovered from a low-quality bin (68% completeness; 5.8% contamination) in the YB3 metagenome (node “ABT-YB3 (low-quality bin)” in Fig. S15a) was divergent (~ 80% ID) from the rest at the nucleotide level, while 95.8% identical to other ABT and *Ca.* Nitrosocosmicus *amoA* genes at the amino acid level. The *rpS3* gene recovered from this bin was classified as thaumarchaeal, with 75% identity to other binned *rpS3* in ABT, and its closest NCBI reference sequence being *Ca.* Nitrosocosmicus sequences at 65% identity. This divergent thaumarchaeal bin was approximately three-fold less abundant than another thaumarchaeal bin (ABT-YB3) recovered at a higher quality from the same metagenome (YB3). Due to the low quality and lower abundance of this divergent bin, our study focuses on other eight mid-to-high quality genomes that are much more closely related and found across all metagenomes under the boulders including YB3.

In order to taxonomically resolve the eight recovered *Thaumarchaeota* genomes, we compared them to *Ca.* Nitrosocosmicus genomes that had been isolated or metagenomically assembled from diverse environments from around the world (Table S7) ranging from the arctic soil [62], tar-contaminated soil [63], vegetable field [64], dinosaur fossil [65] to wastewater filters [66]. High ANI (93.0–99.8%) (Fig. 4c) between ABT genomes indicated that all ABT genomes belong to one genus. Using the ANI threshold of 95% [67, 68] for species delineation, we identified two species within the ABT clade, with genomes recovered from the LB site belonging to one species and the rest to another. The mean amino acid identity (AAI) of 53.9% between pairs of *Ca.* Nitrosocosmicus and ABT genomes (Fig. 4d) fell below the genus delineation threshold of 65% [69] indicating that the two clades form separate genera. Based on these findings, we propose two new species names that belong to a new genus: *Ca.* Nitrosodeserticola atacamae (ABT-LB2, ABT-LB3, ABT-LB) and *Ca.* Nitrosodeserticola subpetralis (ABT-MB1, ABT-MB3, ABT-MB4, ABT-YB1, ABT-YB3). Surveys of the recovered 16S rRNA gene in the NCBI nr database using 99% identity cut-off [70, 71] detected close relatives (no exact matches were found) of the *Ca.* Nitrosodeserticola genomes in diverse locations around the globe such as chromite mine in Iran, alkaline-saline soil of a former lake in Mexico [72], and high-altitude tuff in a Tibetan desert, Armenian hot spring sediment [73], high-elevation soils from a volcano in the Atacama region [74], deglaciation soils in Australia [75], and uranium-contaminated subsurface sediments in the USA [76] (Figure S15b). Our results indicate that close relatives of *Ca.* Nitrosodeserticola are widespread, particularly in soils that experience environmental stress. We also searched for the *Ca.* Nitrosodeserticola *amoA* genes in the NCBI nr database using 97% identity cutoff; however, only one sequence was detected from a Tibetan lake [77] at 97.5% identity.

While the eight *Ca.* Nitrosodeserticola genomes share a highly conserved core genome (mean AAI = 96.5 %), between 11% and 49% (mean = 37.7%) of the genes had no other orthologs in the recovered genomes despite the relatively similar and static environmental conditions that they were found in. High AAI in the orthologous fraction of the eight *Ca.* Nitrosodeserticola genomes and conserved *amoA* sequences recovered in sites more than 200 km apart from each other suggest that these organisms originated from the same strain of *Thaumarchaeota*.

### Pangenomic comparison of ABT genomes and their sister clade reveals unique adaptations including heavy metal resistance, biofilm formation, water transport, and sodium bioenergetics

**Fig. 5:**
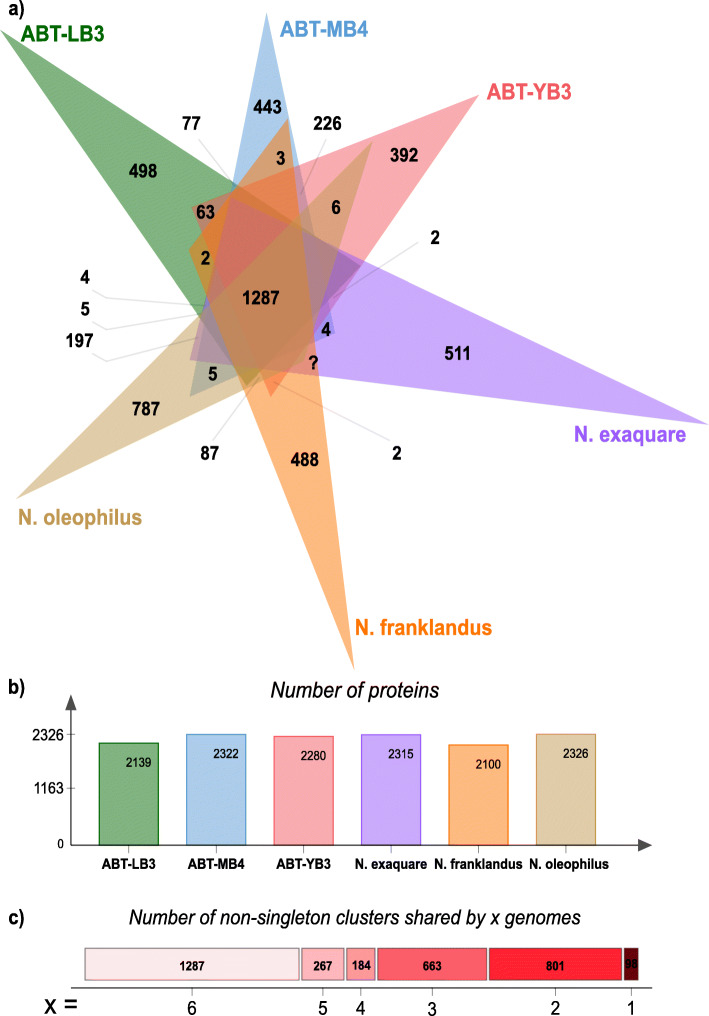
Shared and auxiliary protein clusters of ABT and its sister genus. **a** Shared orthologous protein clusters (including singletons) across six genomes (three *Ca.* Nitrosocosmicus, three *Ca.* Nitrosodeserticola [ABT]). **b** Number of proteins in each genome. **c** Number of orthologous protein clusters (excluding singletons) shared across *x* number of genomes

In order to understand the conserved metabolic potentials between ABT and *Ca.* Nitrosocosmicus, unique adaptations of the ABT in the Atacama Desert, and niche differentiations between sites, we analyzed the high quality (> 95% completeness, <5% contamination) genomes (ABT-LB3, ABT-MB4, ABT-YB3) from each of the three sites along with three (near)-complete *Ca.* Nitrosocosmicus reference genomes (*Ca.* N. franklandus, *Ca.* N. oleophilus, *Ca.* N. hydrocola). 1287 homolog clusters are shared across all six genomes (Fig. 5, Table S7). For example, all genomes contained a highly conserved AmoABX operon, although only two out of eight ABT bins contained 1–2 *amoC* copies. All genomes revealed the metabolic potential for mixotrophy along with important metabolic capacities for nitrogen turnover in these systems, including genes for copper-dependent nitrite reductase (*nirK*), urease (*ureC*), urea transporter, ammonium transporter, deaminases, and lyases for ammonification (Table S8). Additionally, among the shared genes, we found stress response genes such as antioxidant genes (superoxide dismutase and alkyl hydroperoxide reductase), detoxification genes (*cld* and *arsC12* involved in chlorite and arsenate reduction, respectively), as well as bacterial-type DNA repair genes (*radA*, *radB*, *xpf*, *herA*, *nreA*, *uvrABC*) and other resistance genes (*dnaJ*, putative oxidoreductases) (Table S7). These stress resistance genes were found to be conserved and more prevalent in the terrestrial AOA clade than the marine counterparts [78] and a previous study [79] of the terrestrial AOA genome expansion proposed that this extensive suite of stress resistance genes acquired early in the terrestrial AOA evolutionary history provided the basis for their successful colonization of diverse niche terrestrial environments.

We found 296 protein clusters that were shared between ABT genomes but not present in *Ca.* Nitrosocosmicus genomes (Fig. 5). Interestingly, we found extracellular polymeric substances (EPS) and biofilm production genes that were specific to ABT genomes (Table S7). These EPS and biofilm production genes found in the ABT genomes appear to share little homology with genes in *Ca.* N. oleophilus that has previously been shown to form biofilms [63], and suggest recent gain/loss events of the EPS genes following the branching of *Ca.* Nitrosocosmicus and ABT lineages. EPS production and biofilm formation in general are considered major adaptation mechanisms for xerotolerant bacteria [80] and ABT genomes may also employ this mechanism to protect against desiccation. Interestingly, we did not find any genes for the production of compatible solutes (i.e., trehalose, glycine-betaine, ectoine) in any of the ABT genomes, despite it being a well-studied strategy against desiccation and high salt environments [78, 81, 82].

Of the protein-coding genes found in each of the three high-quality ABT genomes, 12.8 to 16.5% belonged to unique protein clusters or were singletons (Fig. 5, Table S9) that did not share any similarity to genes in the other ABT genomes. Among singletons of the genome ABT-LB3, many were involved in membrane transport of metals, such as magnesium, copper, and cobalt transporters as well as lead, cadmium, zinc, and mercury-transporting ATPases, potassium uptake proteins, and bacterioferritin. The presence of these genes may be an adaptation to heavy metals known to accumulate in the Atacama Desert soils [83]. Similarly, notable singletons found in genomes ABT-MB4 and ABT-YB3 included putative cobalt transporter, fluoride transporters, zinc uptake system, mercuric reductase, ferrous iron permease, and phosphite transport system (Please see Supplementary Results R3 for further findings from the pangenome analysis).

Further genome comparisons across all eight ABT genomes revealed additional key adaptations to desiccation and osmotic stress. We identified up to seven different copies of water channel membrane proteins (aquaporin Z2) [84] per genome. Interestingly, some of these proteins were highly divergent from each other at the AA sequence level, while others were truncated (Figure S17) despite being found mid-contig with relatively conserved surrounding genes. Multiple copies of aquaporin genes per genome as well as the divergent and truncated subset indicate possible genome-specific adaptations to desiccation and osmotic stress. We also recovered two distinct types of ATP synthases (namely A-type and V-type [85, 86]) from the eight ABT genomes. Three ABT genomes (ABT-LB2, ABT-LB3, ABT-YB1) contained only A-type ATP synthase, while the rest contained both the A-type and the V-type ATP synthases often in multiple copies. Wang et al. [85] concluded that the V-type ATP synthases were horizontally transferred from *Euryarchaeota* to *Thaumarchaeota* and conserved among the acidophilic and hadopelagic *Thaumarchaeota*, potentially playing a key role in their adaptations to acidic environments and elevated pressure through proton extrusion. Considering that Atacama Desert soils are slightly alkaline (average pH = 7.7 Figure S18), it is surprising that the V-type ATP synthase is found and conserved across five ABT genomes. The hypothesis by Zhong et al. [86] proposing that V-type ATP synthases may be coupled with Na^+^ motive force instead of proton pumping, seems to be a more probable scenario for the ABT. Indeed, Atacama Desert soils present high salt stress, and therefore the V-type ATP synthase could perform Na^+^ pumping and provide protection against high sodium stress (Table S10). In order to understand possible ecological implications of site-specific strains of ABT in soils of varying geochemistry, we created eight genome-based metabolic models using sample-specific geochemical information and ABT genomes binned from the respective samples (see Supplementary Methods M7 for detail) and our results suggest that the geochemical heterogeneity between samples coupled with the flexibility in ABT genomes result in different potentials of N and C flux ratios in each sample, with possible signatures of differentiation by site (Figure S17). Our modeling efforts provide directions for further in situ measurements and experimentation to characterize different levels of C and N turnover by *Ca.* Nitrosodeserticola in the Atacama.

## Conclusions

We report here the first evidence of highly adapted ammonia-oxidizing *Thaumarchaeota* inhabiting the soil beneath the expansive boulder accumulations of the hyperarid Atacama Desert in high relative abundance. This is also the first systematic comparison of microbial communities found below boulders of the Atacama Desert hyperarid core with the microbial communities present in the open, unprotected desert soil. Our study revealed the remarkable adaptability and resilience of *Thaumarchaeota*, expanding the physical limits of thaumarchaeal habitat range to include hyperarid, high-salt, and extremely low-carbon environments. In-depth characterization of these *Ca.* Nitrosodeserticola genomes suggests their potential niche roles in N and C cycling in highly nutrient-deficient Atacama Desert soils, as well as resilience against oxidative stress and hyperaridity. We compared eight closely related *Ca.* Nitrosodeserticola genomes were retrieved from these isolated and disconnected habitats and found that they harbor highly conserved shared genes and large numbers of site-specific auxiliary genes. Our results indicate genomic plasticity of *Ca.* Nitrosodeserticola, whose closest relatives have been found across the globe in terrestrial environments characterized by high oxidative stress and high toxicity. Beyond the Atacama Desert, this study provides a blueprint for future studies of extreme terrestrial environments (i.e., Antarctic and extraterrestrial) where finding pockets of pristine, sheltered, and contained environments, as simple as below boulders, could lead to a discovery of uniquely adapted organisms.

## Material and methods

### Sampling location and procedure

Sampling was conducted in March 2019, during a dry period with the last recorded rain event occurring in June 2017 in the Yungay region. Three sampling sites, Y, M, and L, were chosen based on a previous study [12] that identified inland hyperarid sites using the threshold of water content < 1% by weight (Fig. 1a). The coordinates of the three sample sites can be found in Table S1. Sampling was conducted in previously described characteristic boulder fields [31, 32]. At each boulder field, six boulders of diameter ~ 50 cm and height ~ 20 cm were chosen within a radius of ~100 m from each other. For each boulder, two types of samples were taken: one below boulder (B) and one control sample (C) in the open soil ~ 10 cm away from the boulder (Figure S19), to compare the effect of physical shelter and isolation provided by the boulder on soil microbiomes while minimizing the effect of the spatial heterogeneity in soil geochemistry and mineralogy as observed in previous studies [15, 87]. All chosen boulders were well distanced from other boulders to make sure that the C samples were never constantly shadowed by the sampled boulder or other boulders. In total, 36 samples were collected, twelve (six pairs of B and C samples) across three sampling sites. Samples were collected aseptically from the top 1-cm layer of soil into sterile 50-ml falcon tubes ( ~ 60 g per sample), which were then flash frozen in a liquid nitrogen dry shipper within half an hour of sampling. Control soil samples were taken first and then boulders were flipped over to sample below boulder soil as soon as possible to avoid aerial contamination. Additional samples were taken for geochemical analyses with a small shovel ( ~ 100 g per sample) into a PE-sample bag (Whirl-Pak®, WI, USA) which were then stored at room temperature in the dark. Please see Supplementary Materials and Methods M1 for additional field measurements.

### Geochemical and mineralogical analysis

Detailed methods for pH and electrical conductivity, anion and cation analysis, total organic carbon analysis, and bulk mineralogy can be found in the Supplementary Materials and Methods M2-5.

### Estimation of boulder coverage

Detailed methods for the estimation of boulder field coverage, density, and abundance are described in the Supplementary Materials and Methods M6.

### DNA extraction, Illumina library preparation, and sequencing

Metagenomic DNA was extracted from 10 g of soil as described previously [12]. Briefly, the soil was mixed for 30 minutes in 40 mL of cell extraction buffer (1% PEG 8000; 1M NaCl, pH 9,2) [88]. The supernatant was ultra-centrifuged 2 h at 44,000 × g at 4 °C and DNA was extracted from the pellet using a bead-beating and phenol/chloroform/isoamylalcohol based protocol [89]. DNA was resuspended in 30 μL of DEPC treated water. Two extractions were performed per sample and the resulting DNA was combined. DNA concentration was measured using the Qubit 1x dsDNA HS Assay Kit and Qubit 4 Fluorometer (both Thermo Fisher Scientific, USA). 10 mL of the cell extraction buffer was used as a negative control for DNA extraction. Approximately 5-15 ng of DNA was sheared with a E220 Focused-ultrasonicator (Covaris® Inc., USA), targeting 300–400-bp fragment size, and used to prepare the metagenomic libraries. The libraries were constructed using the NEBNEXT® Ultra II DNA library prep kit for Illumina and the NEBNext® primer set 1 (Dual index, New England BioLabs, UK) with three modifications: 1) the primer adapters were diluted 1:50 v/v, 2) the primers were diluted 1:2 v/v and 3) a second cleaning step was performed after PCR amplification. Purification and size selection were conducted using magnetic beads Agencourt® AMPure® XP (Beckman-Coulter, USA). Inserts between 400 and 500 bp were kept and their quality evaluated using a Fragment Analyzer™ (Advanced Analytical, USA). Library concentration was measured with the Qubit 1x dsDNA HS Assay Kit and Qubit 4 Fluorometer. The metagenomic libraries were sequenced on an Illumina HiSeq 2500 (Illumina, USA) as 2 × 250-bp reads using the HiSeq Rapid SBS Kit v2 (500 cycles, Illumina, USA) and loading 12 pM including 1% v/v PhiX.

### Metagenome assembly, binning, and annotation

Due to low biomass and high levels of inhibitors in the samples, only 15 samples yielded measurable amounts of DNA (Table S2, Figure S20). Of those, eleven DNA extracts successfully yielded metagenomic libraries and subsequent metagenomic analyses were performed. HiSeq reads were quality filtered using BBduk (https://sourceforge.net/projects/bbmap/) and sickle (https://github.com/najoshi/sickle). MetaSPADES 3.13 [90] was used to assemble the reads and the resulting scaffolds were filtered for length (≥ 1000 bp) for gene prediction using Prodigal [91] in meta mode and for annotation using Diamond version 0.9.9 [92] against the UniRef100 database [93] with an *e*-value cut-off of 1E−5. Scaffold coverages were calculated by mapping reads using Bowtie2 in sensitive mode [94]. Genomes were binned using abawaca (github.com/CK7/abawaca), ESOM [95] and MaxBin2 [96], and the resulting bins were aggregated using DAS Tool [97]. Each genomic bin was manually curated using coverage, gene-based taxonomy, and GC content information for each scaffold. ra2 [98] was used to fix assembly errors in all binned scaffolds. CheckM [99] was used to estimate the quality of the bins and only mid-to-high quality bins [100] with completeness > 75% and contamination < 10% were considered for further analysis. For all mid-to-high quality genomes, GTDB-Tk (v1.5.0) classify_wf [101] was used for a broad taxonomic classification and bacterial concatenated protein tree generation. In situ genome replication measures (iRep) [16] and growth rate index (GRiD) [37] were calculated for all bacterial mid-to-high quality genomes. Filtered iRep values (default parameters including genome coverage > 5×) were computed using --mm 3 flag after mapping the reads to scaffolds with Bowtie2 [102]. GRiD values were refined as recommended using the suggested strain heterogeneity threshold (< 0.3). Correlation between the two measures was established using Spearman correlation in R [103] (*p* < 0.03, rho = 0.561, Figure S10b) and iRep was chosen for subsequent analysis and interpretation in this study based on the comparability between the two measures. Further functional and metabolic capacities of mid-to-high quality genomes and metagenomes were determined using METABOLIC [104]. METABOLIC output was further expanded upon using hidden Markov model (HMM) search results for the archaeal AmoA protein (PF12942) (HMMER v3.2.1 (http://hmmer.org/) hmmscan -E 1e−5) and other genes previously annotated using the UniREF100 database [93]. Relative abundances of key metabolic genes were calculated by identifying scaffolds carrying the gene in question, summing up their coverages and then normalizing the summed coverage with the sequencing depth of each sample. 16S rRNA sequences were detected using HMMs (https://github.com/christophertbrown/bioscripts/blob/master/ctbBio/16SfromHMM.py). Hydrogenases were verified and classified using HydDB [105]. RuBisCO sequences were classified using phylogenetic placement. RuBisCO reference sequences were extracted from a local database of dereplicated NCBI genomes (accessed 2019/06/01) with HMMER using the hmm profile (PF00016.22) with 1e−10 *e*-value cutoff. Form IV and most form III RuBisCO sequences were removed and fragmented contiguous sequences were fused. RuBisCO sequences from this study and the Negev desert [55] were added for alignment using MUSCLE [106] followed by BMGE v1.12 trimming [107] with default conditions, and the tree was calculated using IQ-TREE v2.1.2 [108] with flags -m MFP -alrt 1000 -bb 1000.

### Community analysis based on metagenomics

Operational taxonomic units (OTUs) were determined by extracting all genes encoding for the S3 ribosomal protein (*rpS3*) using hmmsearch (HMMER v.3.2.1, http://hmmer.org/) as described previously [109] across all assembled metagenomes. Retrieved RpS3 amino acid sequences were clustered using USEARCH [110] at 99% identity [106] and centroid sequences were extracted. Coverages of OTUs across all samples were calculated by mapping reads from each sample to the scaffolds of the centroids using Bowtie2 in sensitive mode [94] and filtering for a maximum of 5 mismatches (2% error rate) per read for both reads in a read pair. Coverages were then normalized by the total number of reads per sample. OTUs were placed into a phylogenetic tree by aligning using MUSCLE [107], alignment trimming using BMGE [108] in default mode, and tree construction using iqtree v1.3.11.1 [111] with flags -m TEST -alrt 1000 -bb 1000. The phylogenetic tree was visualized using iToL [112]. Shannon-Wiener Indices were calculated using the Vegan package [113] in R [103]. All univariate and multivariate analyses including Bray-Curtis [114] distance matrices for principal coordinate analyses (PCoA), BioENV [38], Non-metric multidimensional scaling (NMDS), and Multiple response permutation procedures (MRPP, permutations = 999) [115] were calculated and subsequently visualized in R v.4.0.2 [103]. Percent relative abundance was calculated using coverage of individual *rpS3* sequences divided by the total coverages of all *rpS3* genes detected in a sample [116].

### Phylogenomic analysis

Phylogenomic placements of the thaumarchaeal metagenome-assembled genomes (MAGs) were determined using a supermatrix of 37 single-copy marker genes with all NCBI genomes annotated as *Thaumarchaeota* as of 4/6/2020 [117]. The fact that the *Thaumarchaeota* classification on NCBI includes the recently reclassified phylum *Aigarchaeota* [118] allowed us to use the latter as an outgroup. CheckM [99] was used to quality filter genomes with thresholds <5% contamination, >50% completeness. Two local databases were created from the Atacama Desert and NCBI *Thaumarchaeota* MAGs (Table S11) respectively. Homologs of Phylosift marker genes [117] were searched in both databases using HMMER 3.2.1 (http://hmmer.org/) with an *e*-value cutoff of 1e−5. The resulting datasets were aligned with MUSCLE with default parameters [107] and curated manually to fuse contiguous fragmented sequences and remove extra gene copies. Ultimately, two genomes (GCA_011605725, GCA_011773305) were removed, since they contained multiple sequences that were too distant from both *Thaumarchaeota* and *Aigarchaeota*, respectively. The resulting datasets were realigned as above, trimmed with BMGE (BLOSUM30) [108], and concatenated into a supermatrix of 312 operational taxonomic units (OTUs) and 7426 positions. Phylogenies were reconstructed with IQ-TREE 2 [111]; first, a tree with ModelFinder [119] (-m MFP -bb 1000 -alrt 1000 -abayes) that served as a guide tree for a run with the PMSF model [120] (-m LG+C60+F+G -bb 1000 -alrt 1000 -abayes). *AmoA* nucleotide sequences were phylogenetically placed using reference AOA genomes with *Ca.* Nitrosocaldus *amoA* as an outgroup [61]. Nucleotide sequence phylogeny for thaumarchaeal 16S rRNA (with references from NCBI nr and SILVA 138.1 Ref NR databases) and amino acid sequence phylogenies for *AmoA* amino acid phylogenetic trees were created as follows: Sequences were aligned using MUSCLE with default parameters [107] and trimmed using BMGE [108] in default mode, and trees we constructed using iqtree v1.3.11.1 [111] with flags -m MFP -alrt 1000 -bb 1000. As per the suggestion of the IQ-TREE authors, we considered those branches strongly supported with at least 95 for ultrafast bootstrap [121] and 80 for the SH-aLRT test [122].

### Comparative genomics

The predicted protein sequences of eight NCBI *Ca.* Nitrosocosmicus reference genomes (Table S3) were compared with the recovered thaumarchaeal MAGs. The CompareM package (github.com/dparks1134/CompareM) was used to identify the orthologous fraction (OF) and calculate the average amino acid identity (AAI) of orthologous genes between a pair of genomes, and fastANI [123] was used to calculate the average nucleotide identity between genomes using default parameters. OrthoVenn2 [124] was used to identify and visualize orthologous clusters across genomes.

### Genome-based metabolic modeling

Detailed methods for the construction of genome-based metabolic models and simulations of carbon uptake and nitrogen output can be found in Supplementary Materials and Methods M7.

## Supplementary Information

**Additional file 1.**

**Additional file 2.**

**Additional file 3.**

**Additional file 4.**

## Data Availability

SRA accession numbers are available in Table S2 and all genomes investigated in this study were deposited to NCBI under BioProject PRJNA665391. Newick treefiles for the concatenated thaumarchaeal tree (Fig. 4), thaumarchaeal 16S rRNA gene tree, and RuBisCO genes are provided in Additional Files 1, 3, and 4 respectively.
