# Supplementary material for: Leave no stone unturned: individually adapted xerotolerant Thaumarchaeota sheltered below the boulders of the Atacama Desert hyperarid core
Source: Microbiome. 2021 Nov 26;9:234. doi: 10.1186/s40168-021-01177-9 (PMC8627038; doi:10.1186/s40168-021-01177-9)
Supplement: Supplementary file 5 — Additional file 4. [file 40168_2021_1177_MOESM5_ESM.zip › Supplementary_Information3.pdf]

Supplementary Information for:

Leave no stone unturned: Individually adapted xerotolerant Thaumarchaeota sheltered below the boulders of the Atacama Desert hyperarid core

Yunha Hwang[1,2,3], Dirk Schulze-Makuch[1,4,5*], Felix L. Arens[1], Johan S. Saenz[6], Panagiotis S. Adam[2], Christof Sager[1], Till L. V. Bornemann[2], Weishu Zhao[7], Ying Zhang[7], Alessandro Airo[1], Michael Schloter[6], Alexander J. Probst[2*]

*corresponding authors

Affiliations:
[1] Astrobiology Group, Center for Astronomy & Astrophysics, Technische Universität Berlin, 10623, Berlin, Germany.
[2] Environmental Microbiology and Biotechnology, Department of Chemistry, University of Duisburg-Essen, 45141, Essen, Germany.
[3] Department of Organismic and Evolutionary Biology, Harvard University, Cambridge, MA, USA
[4] German Research Centre for Geosciences (GFZ), Section Geomicrobiology, 14473 Potsdam, Germany
[5] Department of Experimental Limnology, Leibniz-Institute of Freshwater Ecology and Inland Fisheries (IGB), 12587 Stechlin, Germany
[6] Research Unit for Comparative Microbiome Analysis, Helmholtz Zentrum München, 85758, Oberschleißheim, Germany.
[7] Department of Cell and Molecular Biology, College of the Environment and Life Sciences, University of Rhode Island, Kingston, RI, United States

To whom the correspondence should be addressed:
alexander.j.probst@gmail.com
schulze-makuch@tu-berlin.de

List of Content:

- Supplementary Materials and Methods (M1-M7) - page 2-4
- Supplementary Results and Discussion - page 5-6
- Supplementary Tables S1-2 and Supplementary Figures S1-20 - page 7-32
- Legends for Supplementary Tables S3-12 - page 33
- Descriptions for other Supplementary Files - page 34
- References - page 35-38

Supplementary Materials and Methods

M1. Field measurements

The HOBO U23 pro Temperature/Relative Humidity data logger (Onset, Cat# U23-001, MA, USA) was used to monitor the temperature and relative humidity of each site at the time of sampling. For each sampling site (Y, M, L), an extra boulder was chosen for conducting HOBO logger measurements. One logger was placed under a boulder similarly sized to those chosen for sampling, and another logger was placed ~10 cm away from the boulder on the open soil. For the Y site, a continuous measurement over 130 days (15 March - 25 July 2019) was conducted for characterizing diurnal fluctuations for both below boulder, beside boulder and 1 m above ground. Logged data was then used to calculate dew point temperatures as described in Lawrence *et al.* [1].

M2. pH and electrical conductivity

To evaluate the pH and the electric conductivity (EC) of the soil, samples were prepared in a ratio 1:5 v/v (5 ml sample to 25 ml distilled water), shaken for one hour to prevent the particle from settling and sedimented for another hour, before measuring pH (691 pH Meter, Metrohm, Switzerland). The standard deviation was determined by repeated measurements of in-house standards, SD = 0.24 (n = 16). EC was measured with a handheld electric conductivity meter (GMH 3400, Greisinger, Germany). Reproducibility variation was 5% (n = 3). Both measurements were conducted at the Center of Astronomy and Astrophysics at the Technische Universität Berlin.

M3. Anion and cation analysis

Samples and processing controls for water-soluble ion analysis were prepared based on the standard DIN EN 12457 - 4 (2003) protocol. Briefly, samples were sieved to obtain <2 mm particles which were used to prepare an eluate of a 1:10 w/w (4.5 g sample to 45 g distilled water). After 24 h of continuous shaking, the eluate was filtered through 0.2 µm mesh and stored at -20°C until measurement. Anionic species (F^-, Cl^-, NO_3^-, PO_4^{3-}, SO_4^{2-}) were measured by ion chromatography (DIONEX DX-120 Ion chromatograph, Thermo Fisher Scientific, USA, with a guard column AG 22, 4x50 mm and an analytical column AS 22, 4x250 mm). Reproducibility variation was <1% (n = 5). Cations (Ca^{2+}, Fe^{2+}, K^+, Mg^{2+}, Mn^{2+}, Na^+) were determined by inductively-coupled plasma optical emission spectrometry (iCAP 6000 ICP Spectrometer, Thermo Fisher Scientific, USA). Reproducibility variation was <5% (n = 5). Both analyses were conducted at the Department of Soil Science of the Technische Universität Berlin. Bray-Curtis distance ("vegan" package [2]) metric was used to calculate the distance matrix of samples based on the ion concentrations, which was then used for non-metric multidimensional scaling analysis and environmental vector fitting ("envfit" function, 999 permutations) in R [3]. Paired t-tests and Pearson correlations were performed and p-values corrected for multiple hypotheses testing using Benjamini-Hochberg method were designated as "adjusted p-values".

M4. Total organic carbon analysis

The total organic carbon (TOC) was measured with an elemental analyzer (Vario Max C,

Elementar, Germany) using catalytic tube combustion at the Department of Life Science of the Humboldt Universität Berlin. Samples were first ground to powder. Due to low TOC concentrations, 1 g was used for combustion. At 600°C the organic carbon was removed under the carrier gas nitrogen and oxidized by oxygen in the presence of copper oxide. Remaining elemental carbon was combusted with the addition of oxygen. The resulting CO_2 was then determined successively by infrared detection. The measurement was conducted in duplicates with a detection limit of 0.0124 wt%.

M5. Bulk mineralogy

For the bulk mineralogy, soil samples were homogenized and ground to powder. X-ray powder diffraction (XRD) analysis of the soil salts was performed by using a powder diffractometer (D2 Phaser, Bruker, USA) at the Department of Applied Geochemistry of the Technische Universität Berlin. The X-ray source was Cu Kα radiation (K-alpha1 = 1.540598 Å, K-alpha2 =1.54439 Å) with a performance of 30 kV and 10 mA. A step interval of 0.013° 2Θ with a step-counting time of 0.5 s was used in a scanning range from 3° to 80° 2Θ. Semi-quantitative mineral content was calculated based on relative intensity values using the software package DIFRAC.EVA V2 (Bruker, USA). Absolute reproducibility variation was < 1% (n = 4).

M6. Estimation of boulder coverage.

In this study, boulders are defined by the Wentworth or Udden-Wentworth scale [4,5] as clasts of diameter > 256 mm. Boulder accumulations were mapped at three different resolutions: high, mid and low. 1) For high resolution individual boulder density mapping, drone photography raw data of a 15 m x 180 m transect in the Yungay site (site Y in this study, see **Figure S2a** for the transect location) from Sager *et al.* [6] was reanalyzed. Individual boulders were mapped (see **Figure 1c-d** for an example mapping) and boulder coverage density was calculated. 2) For mid-resolution mapping of boulder accumulations using satellite imagery, study regions were defined as the area within 5 km radius (total area per site: ~78.5 km^2) from the sampling site coordinates (see **Figure S1-2**). Boulder accumulations visible from satellite images (downloaded from Bing Maps) were manually mapped using QGIS [7]. Two categories of boulder accumulations were defined: "densely packed" areas refer to visible continuous boulder accumulations (or "boulder fields" as referred to in this study) where individual boulders could not be distinguished and "loosely packed" areas refer to areas where individual boulders are distinguishable from the typically light sediment background (see the top right corner inset in **Figure S1** for an example satellite image distinguishing the two). The areas where boulder accumulations could not be observed or clearly mapped were distinguished as "non-boulder" areas. 3) For low resolution mapping, we calculated the percent surface area ("intermediate" to "slightly rugged" area at the base of hillslopes) that could harbor boulder accumulations based on a terrain ruggedness index (TRI) (Figure 4b from Kramm *et al.* [8]) map spanning approximately 190,000 km^2 of the Atacama hyperarid core using ImageJ [9].

M7. Genome-scale metabolic modeling of Thaumarchaeal CO_2 uptake and N output

One mid-to-high quality MAG of *Thaumarchaeota* identified from each of the eight below boulder sampling sites, LB2, LB3, LB5, MB1, MB3, MB4, YB1 and YB3, were annotated for

the reconstruction of genome-scale metabolic models (**Additional File 2**). A pangenome analysis was performed on the eight MAGs following prior examples [10] using representative sequences following a 100% identity clustering using CD-HIT [11] at the protein level. To capture metabolic capacities represented by the entire population, representative genes arising from the pangenome analysis were used for the reconstruction of a population-level metabolic model of *Thaumarchaeota*. Annotations of gene-protein-reaction associations were initiated based on homologous mapping to an existing model, NmrFL413, of the *Nitrosopumilus maritimus* SCM1 [12]. Manual curation was performed to incorporate functions that were unique to the MAGs identified from this study, according to the annotations from EggNOG, KEGG, ModelSEED and BIGG. Consistency checks of reactions were performed by using *formulacheck*, *chargecheck*, *dupcheck* and *masscheck* function in PSAMM version 1.0 [11, 12], and curated manually as previously described [13]. Thus, the specific annotation of each MAG from individual sampling sites produced genome-scale models that specifically represent the metabolic capacity of the eight MAGs targeted in our study. Metabolic simulations were performed with exchange constraints identified based on the geochemical measurements of each sampling site, including concentrations of ions and nutrients, such as nitrate, phosphate, and sulfate. Since ammonia concentration was difficult to measure in soil samples, the constraint of ammonia was set based on the nitrogen load identified in the form of nitrate concentrations in each sample, assuming that the conversion of organic matter to ammonia followed by oxidation was the only source of nitrate in the system. To model the correlation of nitrogen and carbon fluxes, the biomass objective function was constrained based on the experimentally estimated concentration of thaumarchaeal biomass in the soil samples (ng dry biomass / g soil), calculated as follows: first, cell counts of *Thaumarchaeota* per sample was estimated using the method previously described by He et al [14]. Estimated cell counts per gram of extracted soil were then multiplied by the dry biomass per cell, which was estimated by direct measurement as 0.0246 pg/cell for a cultured thaumarchaeal AOA strain [15]. Flux variation analysis (FVA) was performed using the *fva* function in PSAMM version 1.0 with the IBM ILOG CPLEX Optimizer version 12.7.1.0 [16,17]. Production of three potential N-products (i.e. N_2O, NO and nitrite) were independently optimized to estimate the maximum capacity of *Thaumarchaeota* from the different samples in producing the different nitrogen species. Uptake ratios of NH_3 and CO_2 were examined in each simulation.

Supplementary Results and Discussion

R1. Boulder coverage estimation

In order to estimate the significance of the below boulder soil compartments as a microbial habitat, we have examined the boulder coverage at three different scales: 1) high resolution using drone photography taken in a previous study [6], 2) mid-resolution with satellite imagery and 3) low-resolution based on a published elevation model [18].

First, we examined the percent of surface soil covered by boulders using the drone photography-based individual boulder mapping raw data from Sager *et al.*[6]. 21.4% and 2.1% of the surface soil were covered by boulders in the "densely" and "loosely" packed sections of the 15 m x 180 m transect (shown in **Figure S2a** in yellow) respectively. The mean boulder size (n = 2492) along the transect was 0.2 m^2 corresponding to approximately 0.51 m in diameter.

We then estimated the coverage of boulder accumulations across the three study sites. Across the mapping area of ~78 km^2 per study site, we approximated the boulder accumulation coverage to be 31% in Yungay (9.6% densely packed, 21.4% loosely packed), 12 % in Maria Elena (7% densely packed, 5% loosely packed) and 16.3% in Lomas Bayas (1% densely packed, 15.3% loosely packed; **Figure S2a-c**). Additionally, the mapping results were consistent with our field observations in that the boulder accumulations are found at the base of hillslopes and at the valley floors of all three study sites. This also agrees with the observations made in previous geomorphic studies of the Atacama Desert and the existing hypothesis that the boulders originate from bedrock outcrops on slopes and hillcrests, and are seismically transported downslope to the base of hills and the valley floors [19,20] (for the boulder fields studied in these papers see **Figure S1**).

Finally, we approximated the potential distribution of landforms that could harbor boulder fields. We estimated the total fraction of the "intermediate" to "slightly rugged" area at the base of hillslopes and valley floors based on Kramm et al (2019)'s terrain ruggedness index (TRI) mapped across ~190,000 km^2 of the Atacama Hyperarid core. Our reanalysis of the TRI figure approximated ~10% of the mapped area (**Figure S3**) to be potential sites where boulders can accumulate.

Based on our analyses of boulder coverage at three different scales, we conclude that 1) the boulders cover a substantial portion (~ 21.4%) of the surface soil in localized dense accumulations (boulder fields), 2) the boulder accumulations are expansive in the regions around our study sites (up to 31% of a circular region of 10 km diameter exhibit boulder accumulations), and 3) the occurrences of expansive boulder accumulations are likely frequent (~10% of the Atacama hyperarid core are potential boulder accumulation sites based on the elevation profile) in other regions of the seismically active hyperarid core of the Atacama Desert. Boulder fields have also been observed in other desert systems such as the Death Valley, USA [21,22], Great Basin Desert, USA [23], the Sonoran Desert, USA [24], Coso Range, USA [25] semiarid to hyperarid deserts on Earth.

R3. Extended pangenome analysis

None of the eight *Thaumarchaeota* genomes contained CRISPR arrays with more than one spacer and only ABT-LB2 contained a putative *cas* gene. Similarly, no *cas* gene was found in previously published genomes of *Ca.* N. franklandus*, Ca.* N. oleophilus and *Ca.* N. hydrocola, and only one *cas* gene was found in *Ca.* N. arcticus. Only *Ca.* N. hydrocola contained an evidence level 4 CRISPR array with 5 spacers (**Table S7**). The lack of CRISPR-Cas systems in these environments could be due to the lower presence of *Thaumarchaeota* targeting viruses in these environments and/or be coupled with slow growth rates rendering the CRISPR-Cas system immune response ineffective [26].

Electron transfer flavoprotein FixABCX genes were found only amongst the ABT genomes and not in any of *Ca.* Nitrosocosmicus (**Table S7**). These genes are reported to be involved in the electron bifurcation in diazotrophs [27], but are also found in many non-diazotrophic *A*rchaea [28], where their function is yet to be determined.

No S-layer protein slp1 was found in any of the ABT nor other *Ca*. Nitrosocosmicus. However, we detected hexuronic acid methyltransferase AglP in ABT genomes, which is involved in the pathway of S-layer biogenesis, suggesting that there may exist an alternative pathway for S-layer production. This could provide additional protection against harsh desert environments for the ABT genomes [29].

Notably, all genomes featured high-affinity Na^+/H^+ antiporter NhaS, with ABT-LB2 and ABT-LB3 genomes featuring five copies, while the others featured a single copy. This may be correlated to the lack of Na^+ binding V-type ATP synthase in ABT-LB2 and ABT-LB3 genomes. Additional genes associated with Na^+ bioenergetics were identified, including sodium/glucose transporter, putative calcium/sodium:proton antiporter, sodium bile acid symporter family protein, sodium/hydrogen exchanger and sodium-dependent dicarboxylate transporters. High number of sodium bioenergetics genes suggests that ABT genomes are adapted to high salt concentrations and may also be capable of utilizing the sodium gradient for scavenging biomolecules and ATP generation.

Supplementary Figures and Tables

Table S1: Sampling information, temperature and relative humidity below and beside boulders were measured using OBO U23 pro temperature/relative humidity data logger at the time of sampling.

Site	Location Name	Longitude	Latitude	Altitude (m above sea level)	Temp Below (°C)	Temp Beside (°C)	Relative Humidity Below (%)	Relative Humidity Beside (%)	Collection Time (+/- 15 min)	Collection Date
**Y**	Yungay	-69.99927	-24.08663	1067	22.29	33.55	31.81	21.24	11:00:00 AM	10/3/2019
**M**	Maria Elena	-69.72428	-22.26319	1318	18.6	14.3	32.6	68.4	8:30:00 AM	13/3/2019
**L**	Lomas Bayas	-69.60378	-23.39321	1521	36.25	37.53	11.91	13.71	12:30:00 PM	13/3/2019

Table S2. Metagenome library information. DNA extracts from MB6, MC3, YB2 and YB5 contained measurable DNA, yet failed in library preparation (see **Figure S18**).

Library	# Read pairs	Assembly size (bp)	# scaffolds	# scaffolds >=1kbp	N50 (scaffolds >=1kbp)	Sequencing depth	NCBI Accession ID
LB2	22,130,842	303,328,226	529,525	53,921	2,515	9,768,467,534	SAMN16268202
LB3	31,273,647	388,980,799	619,007	73,975	2,915	13,479,472,613	SAMN16268203
LB5	42,100,542	27,9268,968	502,503	46,030	2,262	18,239,942,017	SAMN16268204
LC2	41,952,484	390,674,555	678,127	74,711	3,212	16,737,394,618	SAMN16268205
LC3	20,973,878	385,836,147	692,036	72,209	2,639	9,268,440,081	SAMN16268206
LC5	36,938,861	498,167,674	1,001,606	80,101	2,521	15,452,539,821	SAMN16268207
MB1	50,912,651	397,090,821	724,094	64,247	1,961	21,951,582,505	SAMN16268208
MB3	13,796,379	325,366,862	602,775	55,520	1,895	5,836,722,551	SAMN16268209
MB4	36,370,037	490,933,896	960,960	74,205	2,119	16,170,303,555	SAMN16268210
YB1	15,371,532	295,824,573	499,610	54,472	2,284	6,539,418,086	SAMN16268211
YB3	27,738,528	335,124,123	571,894	58,118	2,245	12,052,827,536	SAMN16268212

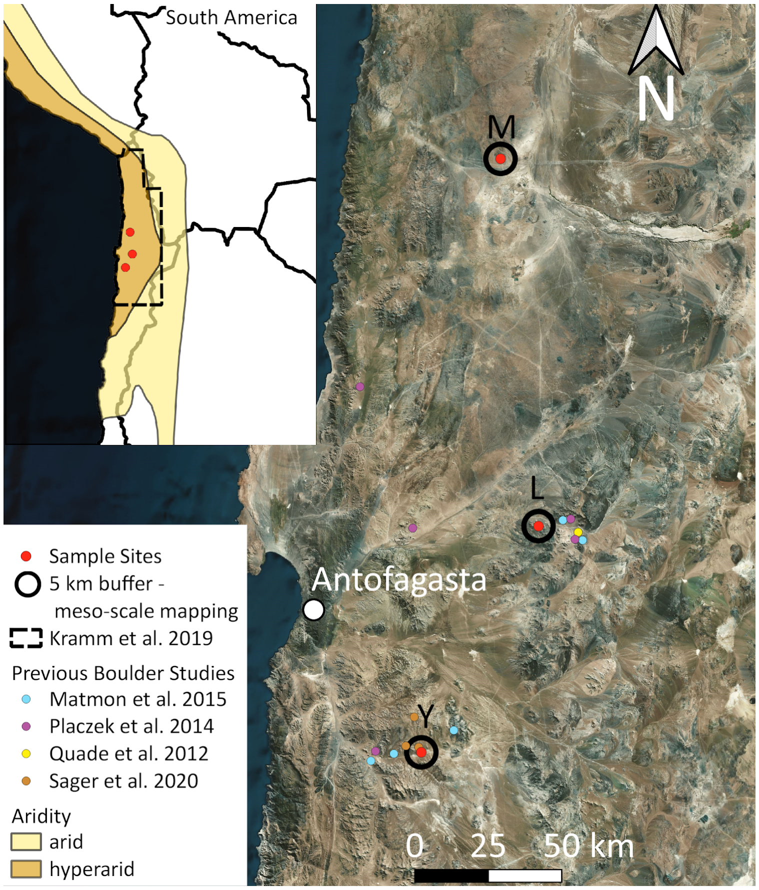

Figure S1. Satellite image showing the locations of the boulder accumulation coverage analyses. Circles outline the regions where the mid-resolution boulder accumulation mapping was conducted. Locations of previous boulder studies are noted with color-coded points. Dashed line in the top-left inset marks the total area analyzed in low-resolution mapping of potential boulder accumulation shown in **Figure S3**. Arid and hyperarid climate regions are mapped according to Houston and Hartley [30].

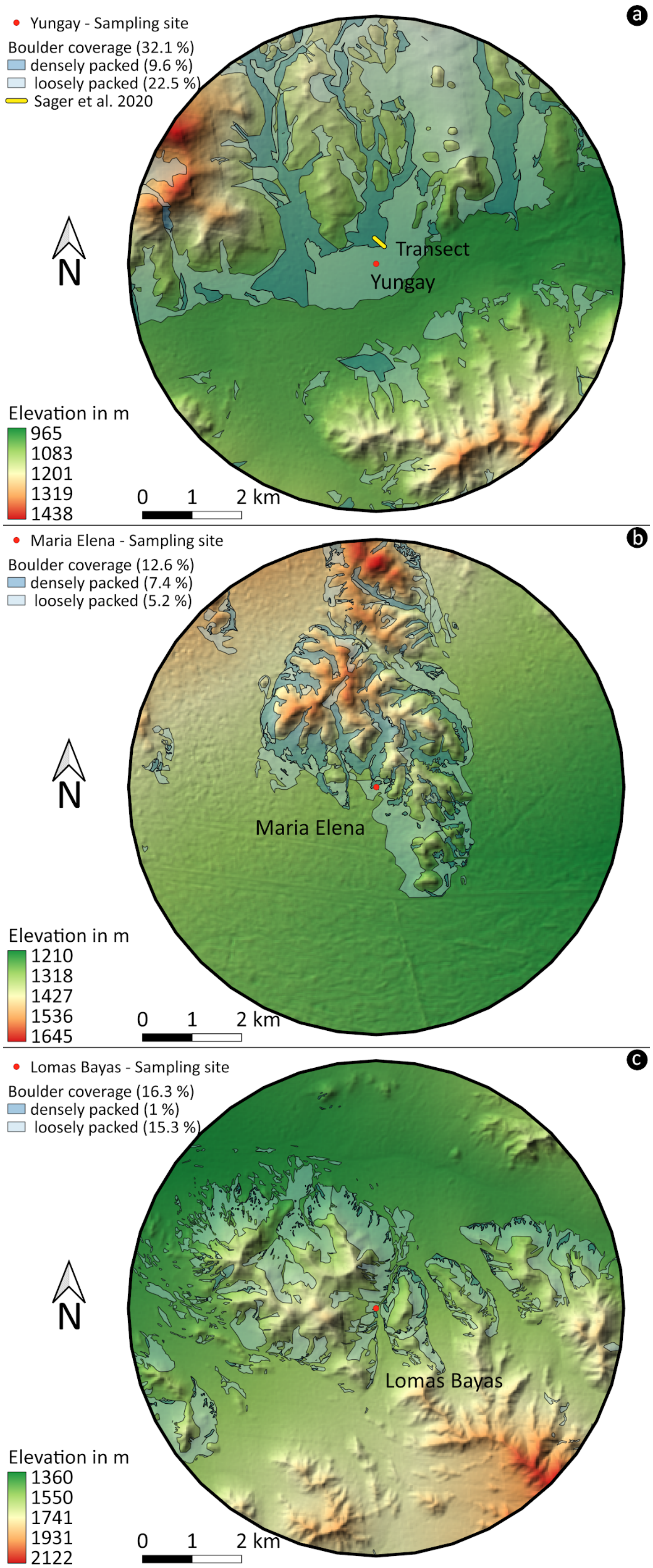
a
Yungay - Sampling site
Boulder coverage (32.1 %)
densely packed (9.6 %)
loosely packed (22.5 %)
Sager et al. 2020
Transect
Yungay
N
Elevation in m
965
1083
1201
1319
1438
0 1 2 km
b
Maria Elena - Sampling site
Boulder coverage (12.6 %)
densely packed (7.4 %)
loosely packed (5.2 %)
N
Maria Elena
Elevation in m
1210
1318
1427
1536
1645
0 1 2 km
c
Lomas Bayas - Sampling site
Boulder coverage (16.3 %)
densely packed (1 %)
loosely packed (15.3 %)
N
Lomas Bayas
Elevation in m
1360
1550
1741
1931
2122
0 1 2 km

Figure S2. Shaded digital elevations model showing the mapped boulder accumulations (mid-resolution) of the study regions (a: Yungay [Y], b: Maria Elena [M], c: Lomas Bayas [L]). Red dot marks the sampling location at the center of the mapped circular region (5 km radius). Yungay site (a) shows the transect used for high-resolution mapping of boulders. An example of “densely” and “loosely” packed boulder accumulations as seen in a satellite image is shown in **Figure 1e**.

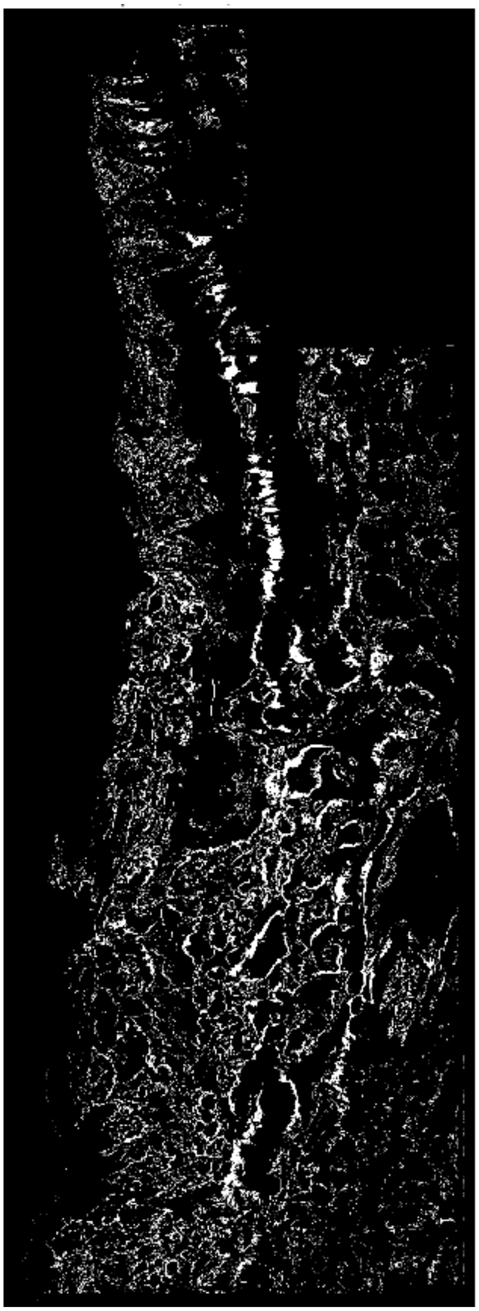

Figure S3. Analysis of terrain ruggedness index (TRI) map (Figure 4b) in Kramm *et al.* (2019) [18]**.** White pixels mark the "intermediate" to "slightly rugged" area at the base of hillslopes. Image reanalysis was conducted using ImageJ [9].

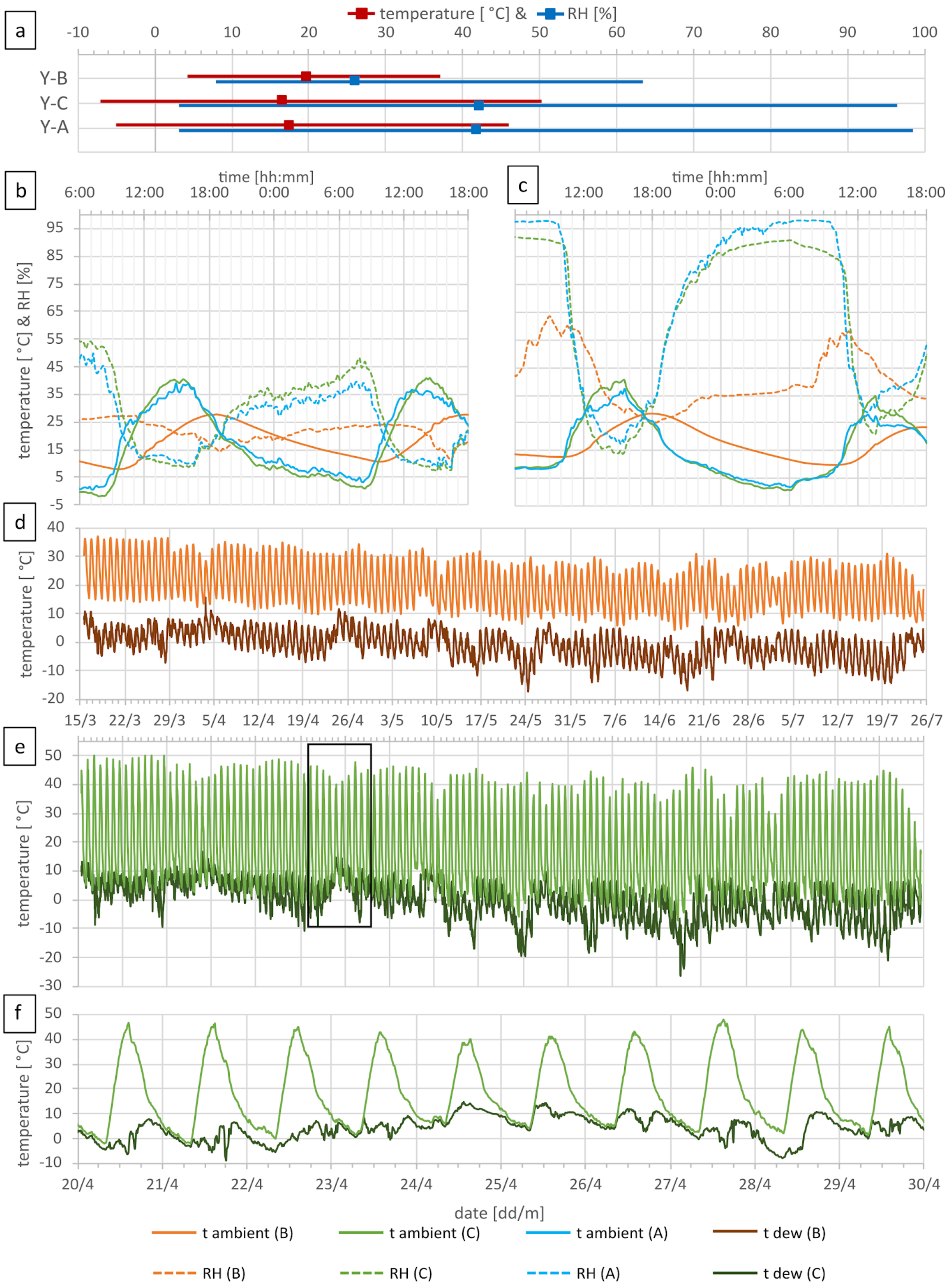

Figure S4. Extended field measurements of temperature and relative humidity (RH) for Y-below boulder (B), control (C) and at 1 m above ground (A). a) Mean temperature and RH (square) and range (bar). b) Temperature and RH during a dry diurnal cycle; c) Temperature and RH during a moist diurnal cycle. d) Ambient temperature and calculated dew point temperature below boulder (B) during the full 130 days of recording. e) Ambient temperature and calculated

dew point temperature beside boulder (control) during the full 130 days of recording, black rectangle is zoomed in panel f).

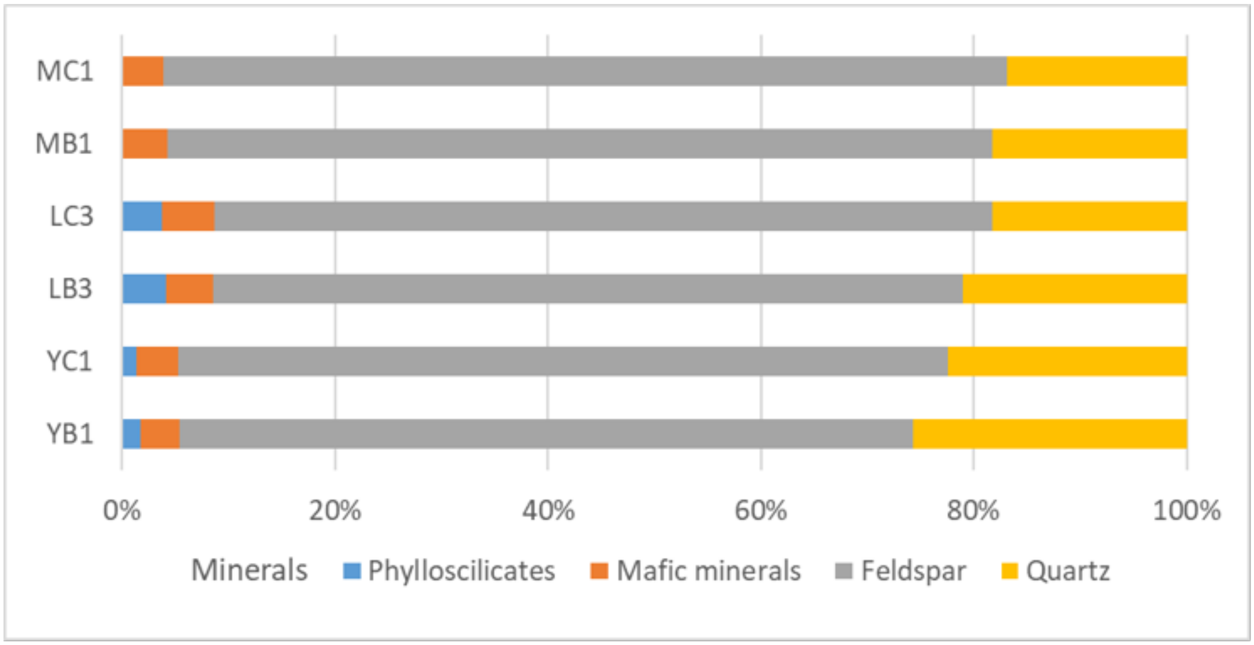

Figure S5. Mineral composition (wt %) determined using XRD analysis.

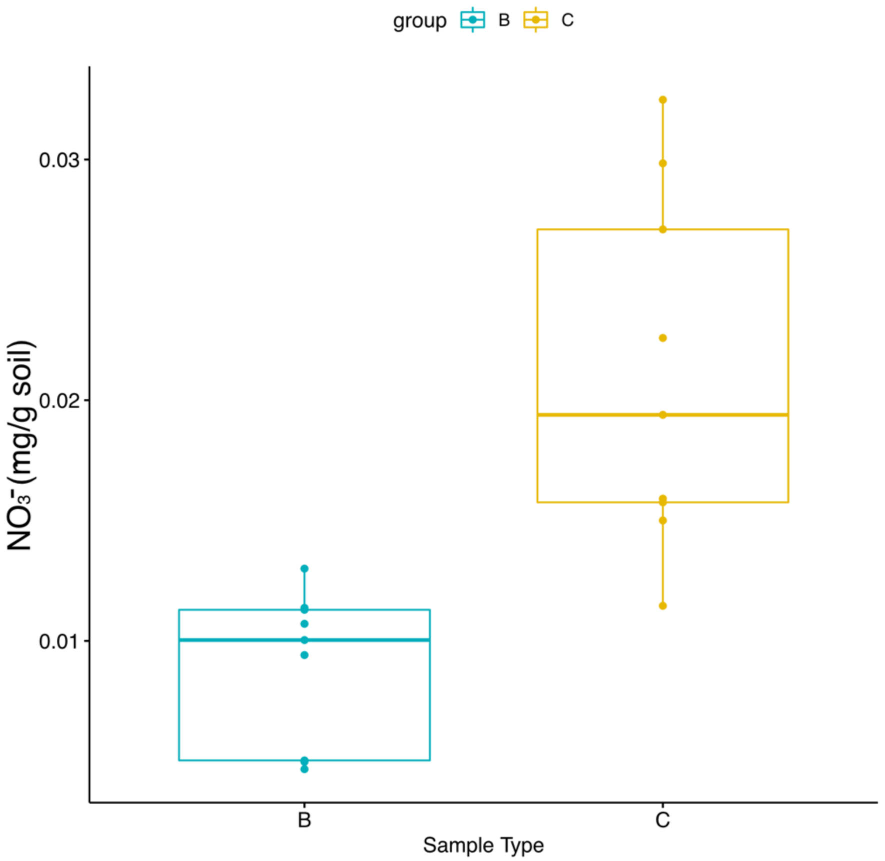

Figure S6. Comparison of nitrate ion concentrations between below boulder (B) and beside boulder control (C) sample types. Plot was visualized using "ggpubr" package in R.

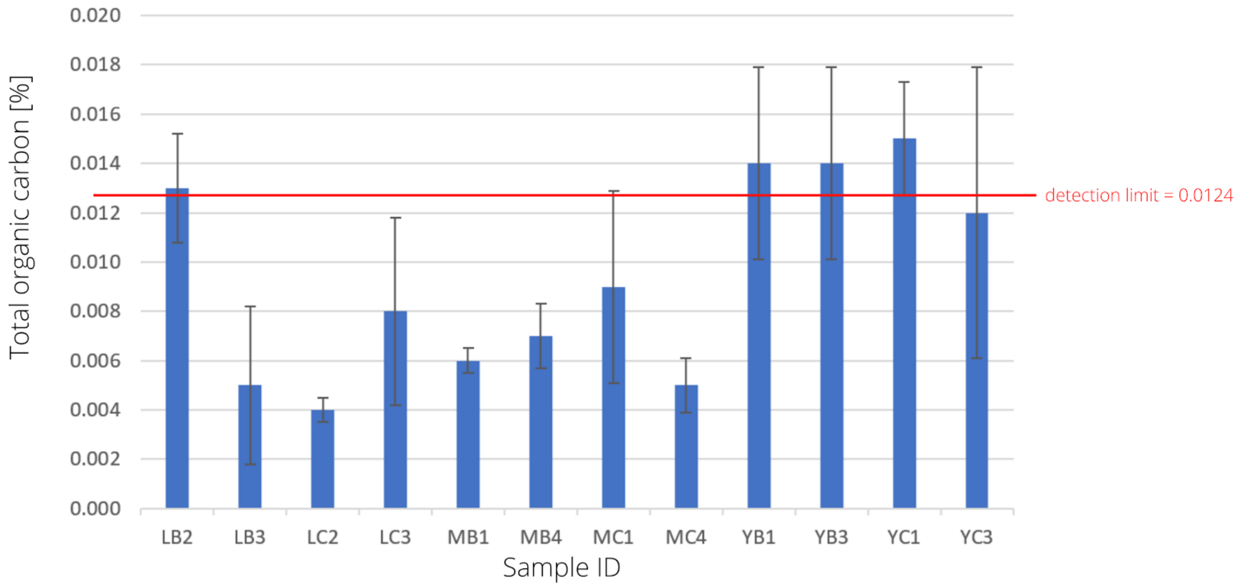

Figure S7. Total Organic Carbon (TOC) content [wt%] per sample. All values were below the limit of quantitation value 0.02962 wt% and very close to or below the limit of detection 0.0124 wt%.

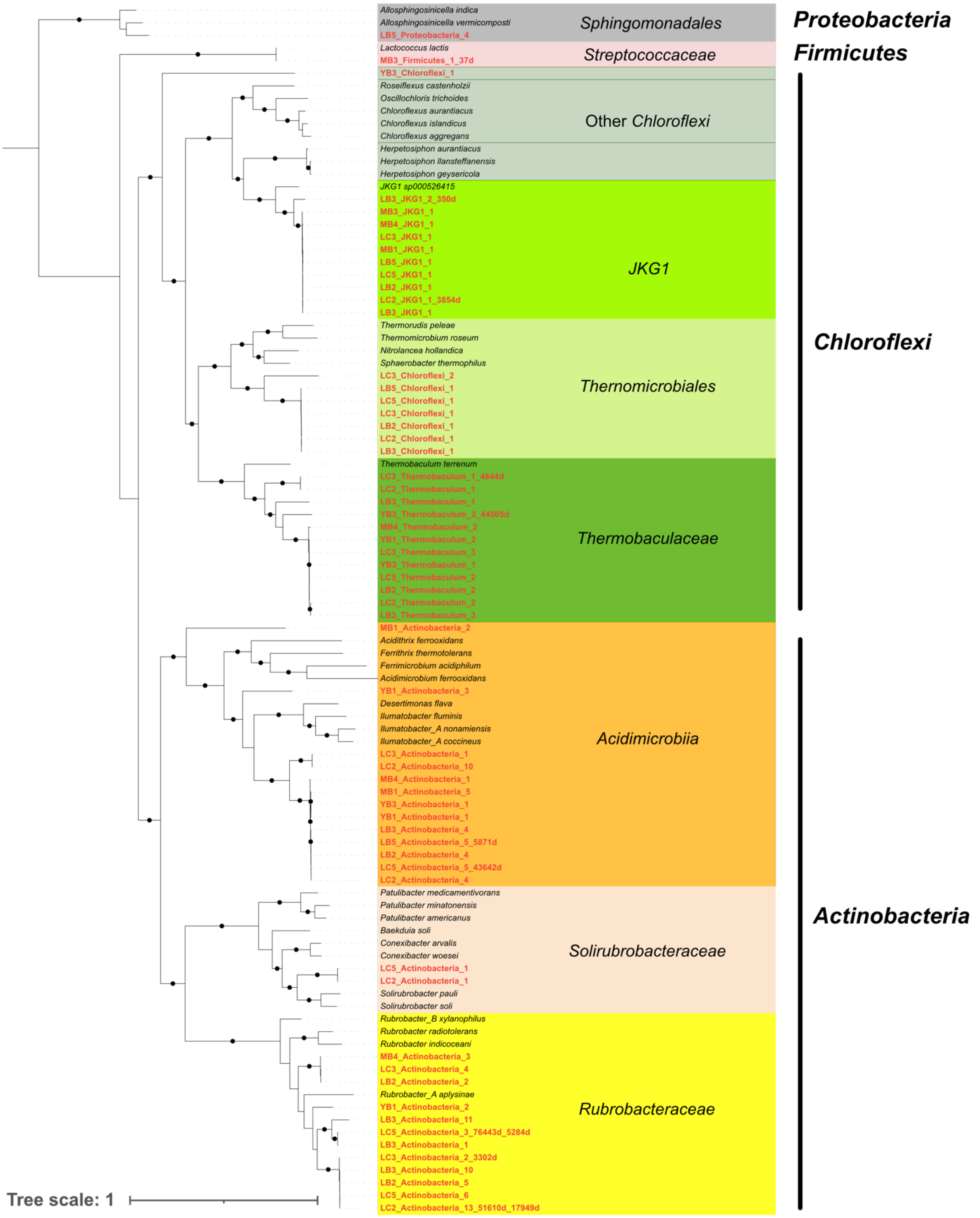
Allosphingosinicella indica
Allosphingosinicella vermicomposti
LB5_Proteobacteria_4
Sphingomonadales
Proteobacteria
Lactococcus lactis
MB3_Firmicutes_1_37d
Streptococcaceae
Firmicutes
YB3_Chloroflexi_1
Roseiflexus castenholzii
Oscillochloris trichoides
Chloroflexus aurantiacus
Chloroflexus islandicus
Chloroflexus aggregans
Other Chloroflexi
Herpetosiphon aurantiacus
Herpetosiphon llansteffanensis
Herpetosiphon geysericola
JKG1 sp000526415
LB3_JKG1_2_350d
MB3_JKG1_1
MB4_JKG1_1
LC3_JKG1_1
MB1_JKG1_1
LB5_JKG1_1
LC5_JKG1_1
LB2_JKG1_1
LC2_JKG1_1_3854d
LB3_JKG1_1
JKG1
Chloroflexi
Thermorudis peleae
Thermomicrobium roseum
Nitrolancea hollandica
Sphaerobacter thermophilus
LC3_Chloroflexi_2
LB5_Chloroflexi_1
LC5_Chloroflexi_1
LC3_Chloroflexi_1
LB2_Chloroflexi_1
LC2_Chloroflexi_1
LB3_Chloroflexi_1
Thernomicrobiales
Thermobaculum terrenum
LC3_Thermobaculum_1_4844d
LC2_Thermobaculum_1
LB3_Thermobaculum_1
YB3_Thermobaculum_3_44505d
MB4_Thermobaculum_2
YB1_Thermobaculum_2
LC3_Thermobaculum_3
YB3_Thermobaculum_1
LC5_Thermobaculum_2
LB2_Thermobaculum_2
LC2_Thermobaculum_2
LB3_Thermobaculum_3
Thermobaculaceae
MB1_Actinobacteria_2
Acidithrix ferrooxidans
Ferrithrix thermotolerans
Ferrimicrobium acidiphilum
Acidimicrobium ferrooxidans
YB1_Actinobacteria_3
Desertimonas flava
Ilumatobacter fluminis
Ilumatobacter_A nonamiensis
Ilumatobacter_A coccineus
LC3_Actinobacteria_1
LC2_Actinobacteria_10
MB4_Actinobacteria_1
MB1_Actinobacteria_5
YB3_Actinobacteria_1
YB1_Actinobacteria_1
LB3_Actinobacteria_4
LB5_Actinobacteria_5_5871d
LB2_Actinobacteria_4
LC5_Actinobacteria_5_43642d
LC2_Actinobacteria_4
Acidimicrobiia
Patulibacter medicamentivorans
Patulibacter minatonensis
Patulibacter americanus
Baekduia soli
Conexibacter arvalis
Conexibacter woesei
LC5_Actinobacteria_1
LC2_Actinobacteria_1
Solirubrobacter pauli
Solirubrobacter soli
Solirubrobacteraceae
Actinobacteria
Rubrobacter_B xylanophilus
Rubrobacter radiotolerans
Rubrobacter indicoceani
MB4_Actinobacteria_3
LC3_Actinobacteria_4
LB2_Actinobacteria_2
Rubrobacter_A aplysinae
YB1_Actinobacteria_2
LB3_Actinobacteria_11
LC5_Actinobacteria_3_76443d_5284d
LB3_Actinobacteria_1
LC3_Actinobacteria_2_3302d
LB3_Actinobacteria_10
LB2_Actinobacteria_5
LC5_Actinobacteria_6
LC2_Actinobacteria_13_51610d_17949d
Rubrobacteraceae
Tree scale: 1

Figure S8. Phylogeny of mid-to-high quality bacterial MAGs and reference genomes. Phylogenetic marker genes (n=120) were identified, concatenated and aligned using GTDB-Tk [31] and the tree was calculated using IQ-TREE v2.1.2 [32] with flags -m MFP -alrt 1000 -bb 1000 and visualized using iToL[33]. Color codes for taxonomic groups are identical to figures 2 and 3. Branches marked with black circles are considered strongly supported (SH-alrt test value > 80, bootstrap value > 95)

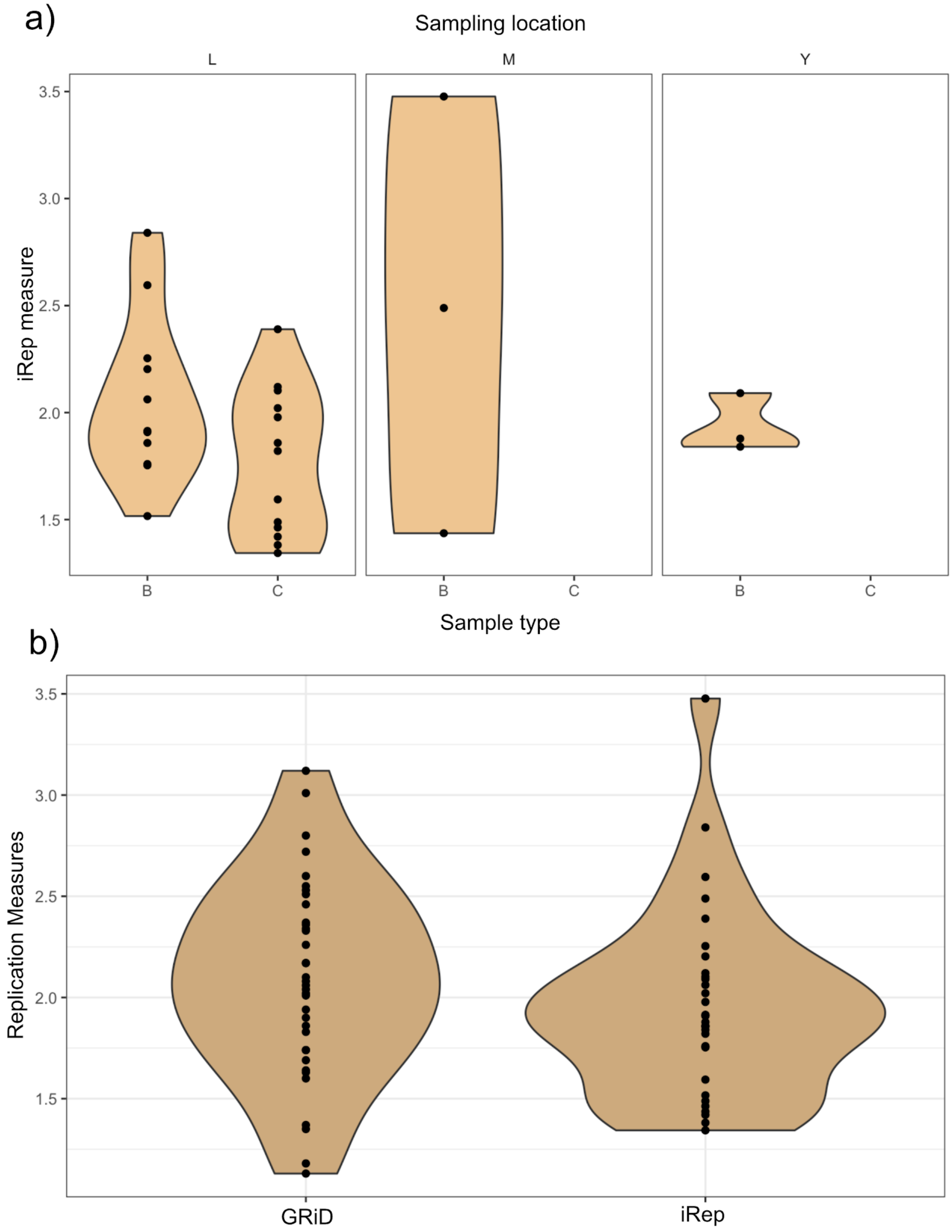

Figure S9. a) calculated iRep values of genomes of sample type (B and C) and sample sites (L, M and Y). b) comparison of iRep and GRiD. Refined GRiD values (n=36) and filtered iRep values (n=30) are depicted. Spearman correlation indicated a significant correlation of iRep and GRiD values on the genomes for which both measures could be calculated ($p < 0.03$, rho=0.561). Plots were visualized using ggplot2 [34].

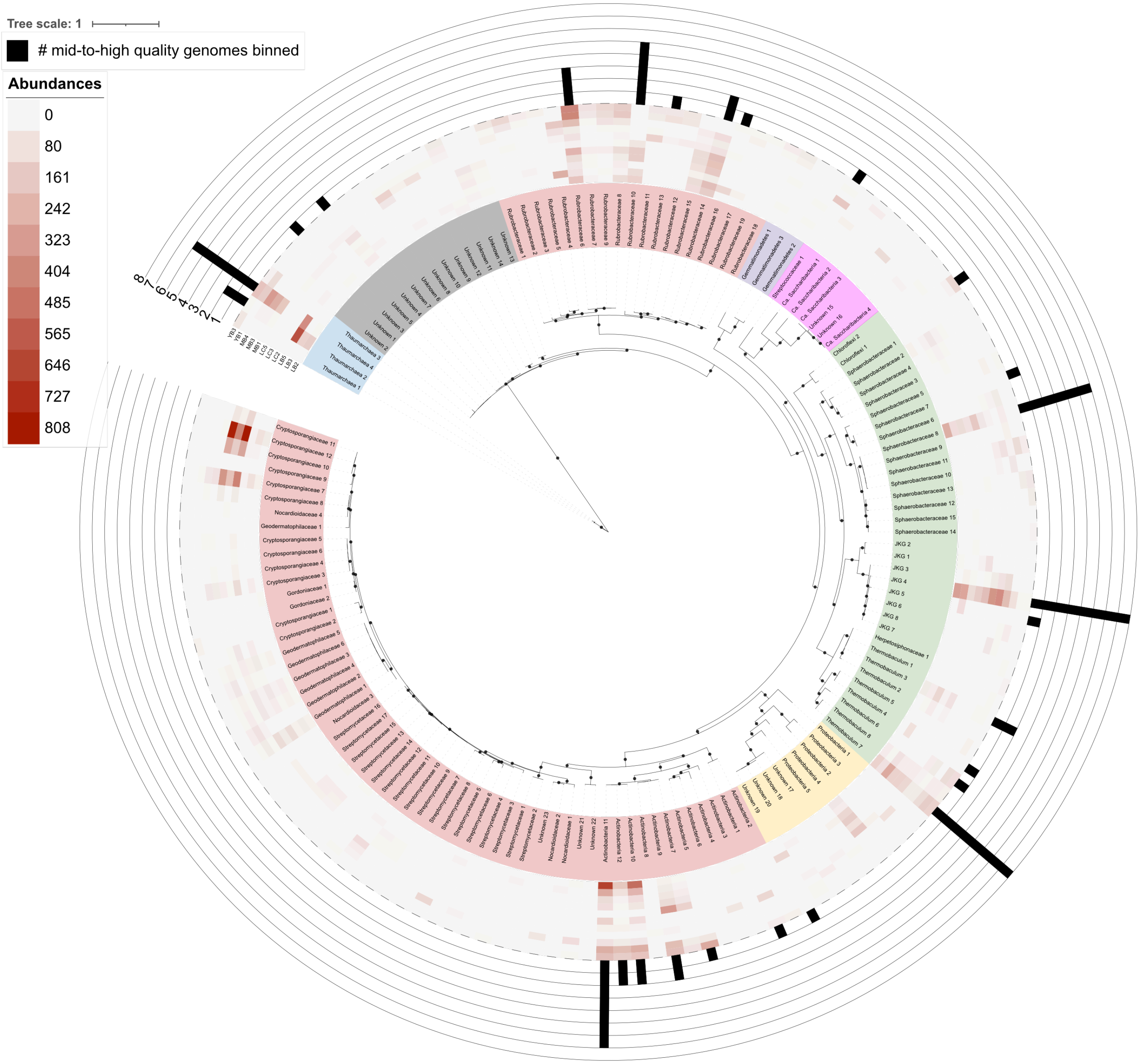

Figure S10. Full phylogenetic tree of all recovered rpS3 gene clusters. Color ranges refer to phyla level classification, leaf labels refer to taxonomic resolution down to family level based on BLAST [35] results against UniRef100 [36]. Alignment was made using MUSCLE [37] followed by BMGE v1.12 trimming [38] with default conditions, and the tree was calculated using IQ-TREE v1.5.5 [32] with flags -m MFP -alrt 1000 -bb 1000. Visualization was done using iToL [33] and branches marked with black circles are considered strongly supported (SH-alrt test value > 80, bootstrap value > 95). Abundances across the samples are shown as a heatmap and the number of the binned mid-to-high quality genomes are displayed as a bar chart for each gene cluster node.

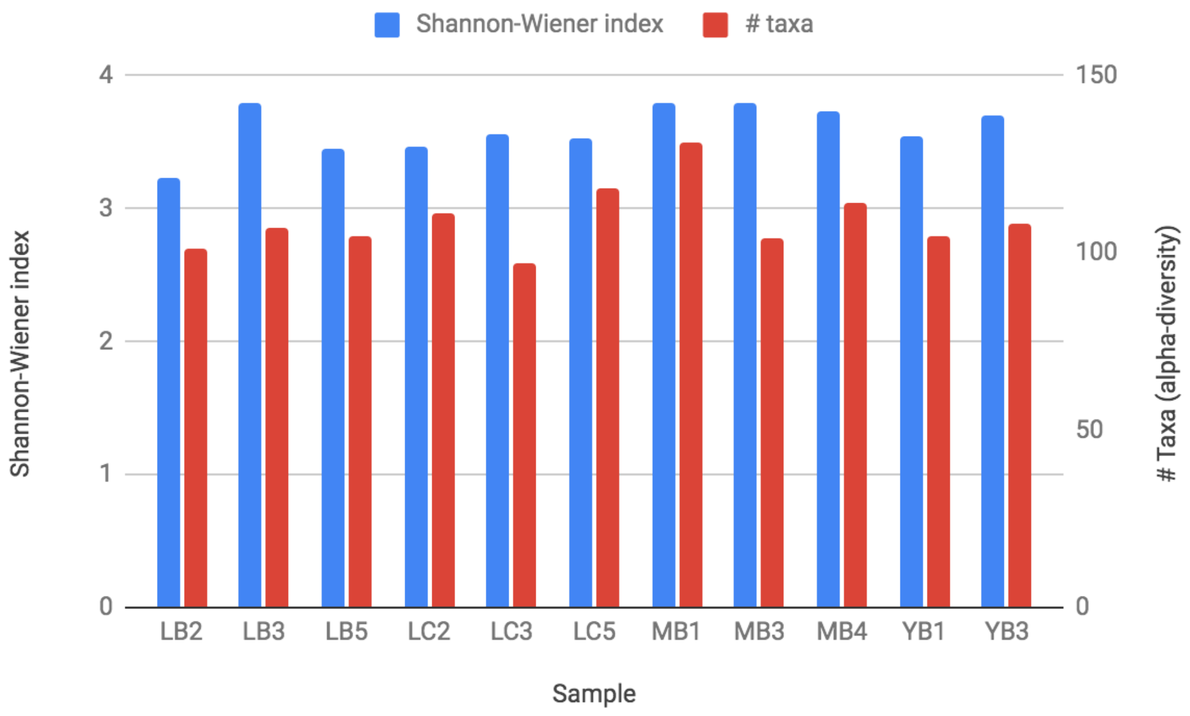

Figure S11. Shannon-Wiener index of each metagenome. Indices were calculated based on normalized ribosomal protein S3 (rpS3) abundances.

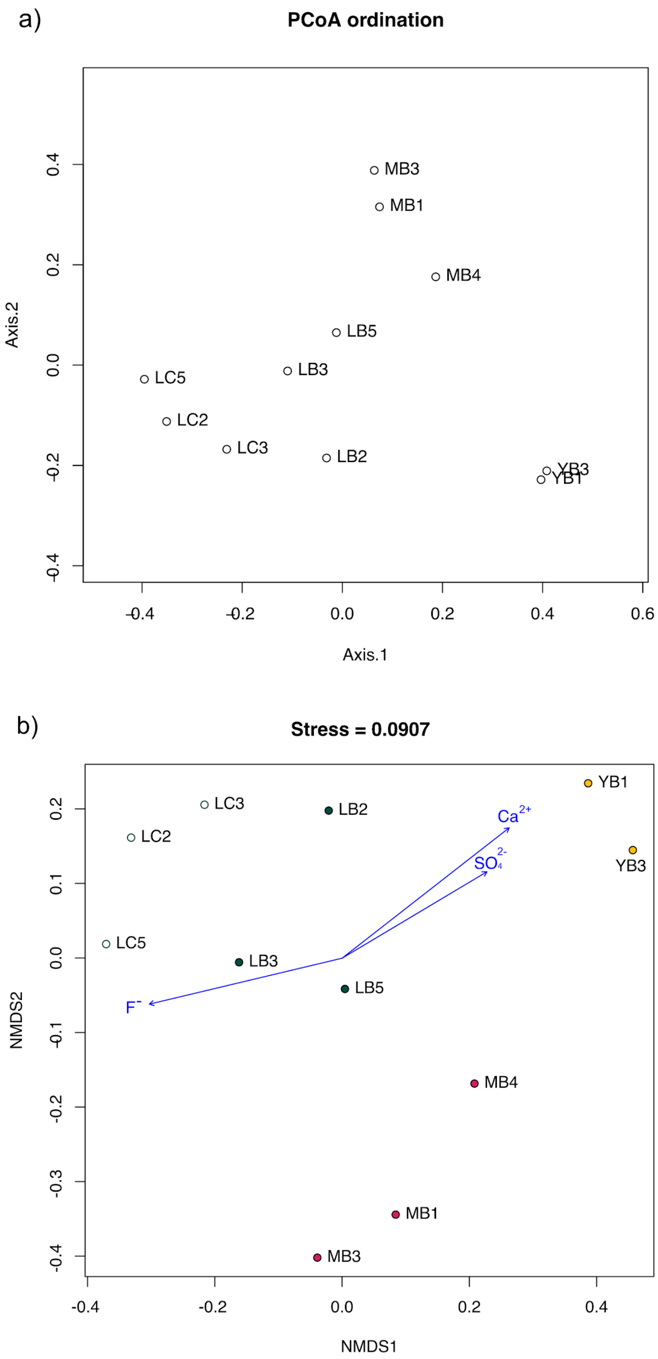

Figure S12. Principal Coordinate Analyses (PCoA) of microbial communities. a) PCoA (Explained variance axis 1: 0.366, Explained variance axis 2: 0.235, Explained for both axis: 0.600) and b) NMDS ordination plots of metagenomes based on rpS3 abundances. Bray-Curtis distance matrix of normalized abundances of rpS3 taxa across all metagenomes were calculated and used as input for both figures. For b) ion concentrations for each sample were added and the corresponding vectors were fitted with the NMDS ordination. Blue arrows represent fitted ion species with a p-value less than 0.05. Both figures were generated using R [3].

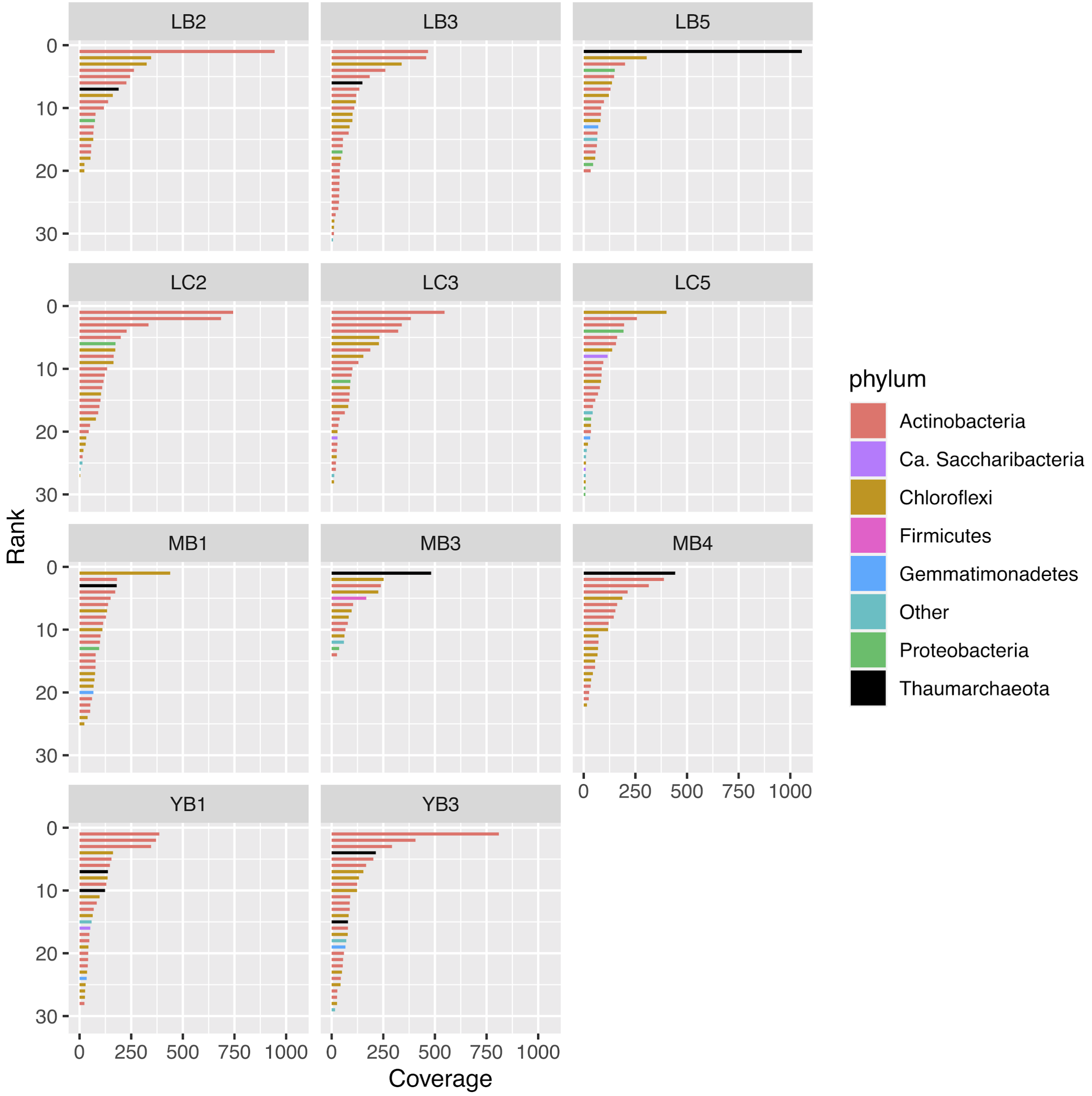

Figure S13. Rank abundance of *rpS3* genes based on normalized coverage. Thaumarchaeal *rpS3* rank abundances highlighted with color black. Coverages were normalized across the samples using the total number of reads (sequencing depth) of each sample metagenome. Visualization was done using ggplot2 [34] in R [3] version 4.0.2 (2020-06-22).

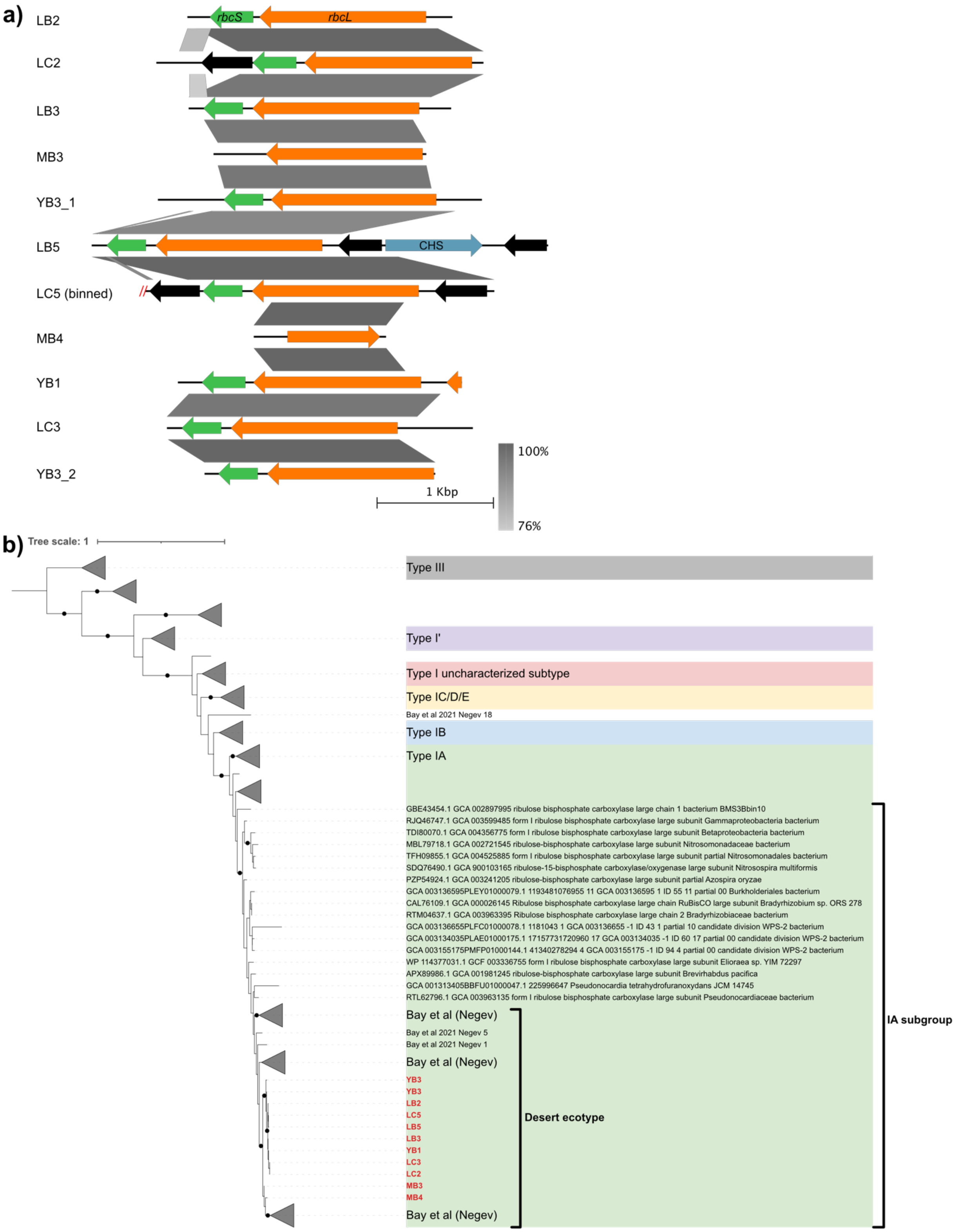
a)
LB2
rbcS
rbcL
LC2
LB3
MB3
YB3_1
LB5
CHS
LC5 (binned)
MB4
YB1
LC3
YB3_2
100%
76%
1 Kbp
b)
Tree scale: 1
Type III
Type I'
Type I uncharacterized subtype
Type IC/D/E
Bay et al 2021 Negev 18
Type IB
Type IA
GBE43454.1 GCA 002897995 ribulose bisphosphate carboxylase large chain 1 bacterium BMS3Bbin10
RJQ46747.1 GCA 003599485 form I ribulose bisphosphate carboxylase large subunit Gammaproteobacteria bacterium
TDI80070.1 GCA 004356775 form I ribulose bisphosphate carboxylase large subunit Betaproteobacteria bacterium
MBL79718.1 GCA 002721545 ribulose-bisphosphate carboxylase large subunit Nitrosomonadaceae bacterium
TFH09855.1 GCA 004525885 form I ribulose bisphosphate carboxylase large subunit partial Nitrosomonadales bacterium
SDQ76490.1 GCA 900103165 ribulose-15-bisphosphate carboxylase/oxygenase large subunit Nitrosospira multiformis
PZP54924.1 GCA 003241205 ribulose-bisphosphate carboxylase large subunit partial Azospira oryzae
GCA 003136595PLEY01000079.1 1193481076955 11 GCA 003136595 1 ID 55 11 partial 00 Burkholderiales bacterium
CAL76109.1 GCA 000026145 Ribulose bisphosphate carboxylase large chain RuBisCO large subunit Bradyrhizobium sp. ORS 278
RTM04637.1 GCA 003963395 Ribulose bisphosphate carboxylase large chain 2 Bradyrhizobiaceae bacterium
GCA 003136655PLFC01000078.1 1181043 1 GCA 003136655 -1 ID 43 1 partial 10 candidate division WPS-2 bacterium
GCA 003134035PLAE01000175.1 17157731720960 17 GCA 003134035 -1 ID 60 17 partial 00 candidate division WPS-2 bacterium
GCA 003155175PMFP01000144.1 41340278294 4 GCA 003155175 -1 ID 94 4 partial 00 candidate division WPS-2 bacterium
WP 114377031.1 GCF 003336755 form I ribulose bisphosphate carboxylase large subunit Elioraea sp. YIM 72297
APX89986.1 GCA 001981245 ribulose-bisphosphate carboxylase large subunit Brevirhabdus pacifica
GCA 001313405BBFU01000047.1 225996647 Pseudonocardia tetrahydrofuranoxydans JCM 14745
RTL62796.1 GCA 003963135 form I ribulose bisphosphate carboxylase large subunit Pseudonocardiaceae bacterium
Bay et al (Negev)
Bay et al 2021 Negev 5
Bay et al 2021 Negev 1
Bay et al (Negev)
YB3
YB3
LB2
LC5
LB5
LB3
YB1
LC3
LC2
MB3
MB4
Bay et al (Negev)
Desert ecotype
IA subgroup

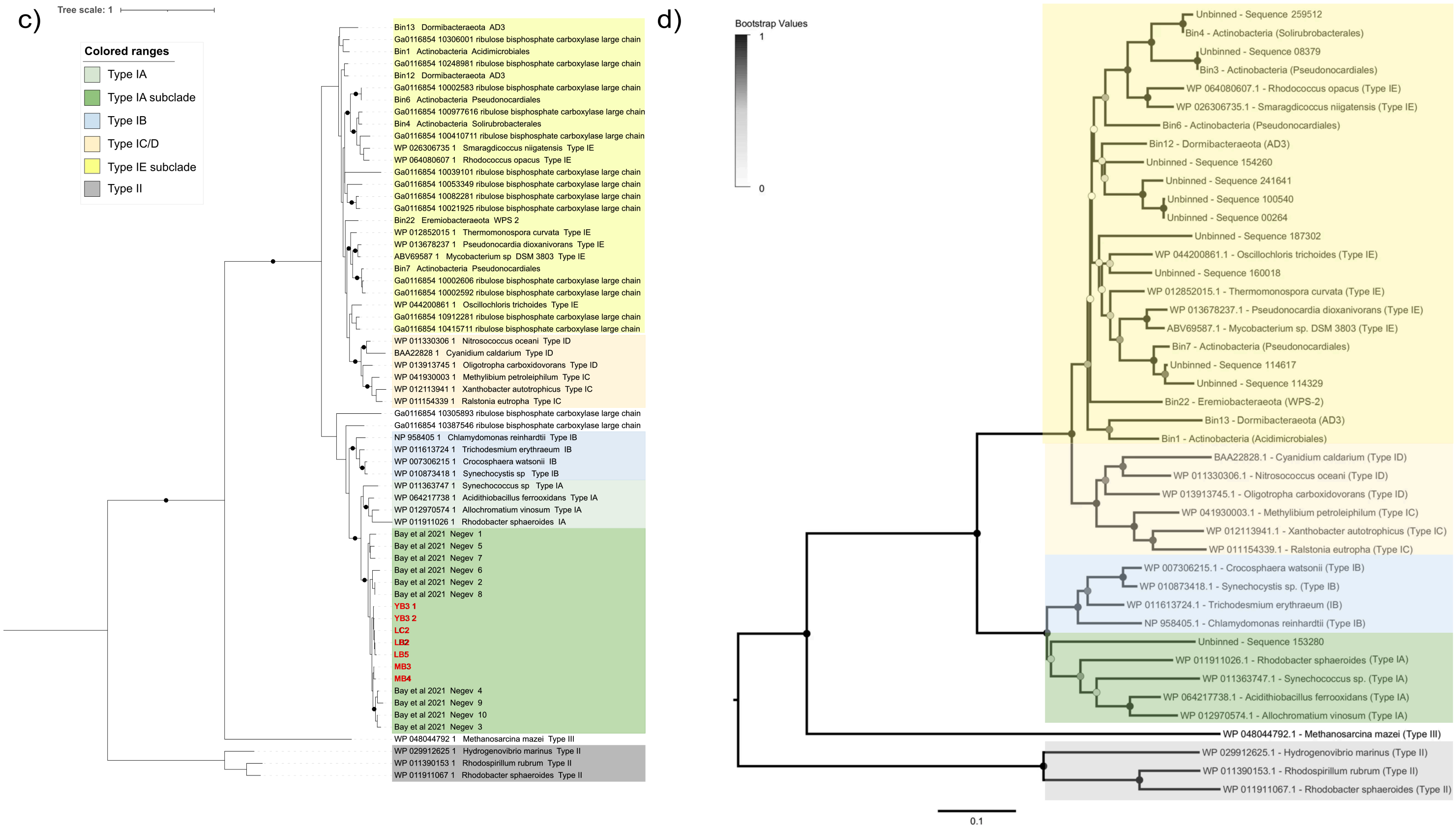

Figure S14. RuBisCO contigs and phylogeny. a) Contigs featuring RuBisCO large (orange) and small (green) subunits. Only one contig from LC5 metagenome were assembled into a longer (64kbp) scaffold which could be binned into an *Acidimicrobiia* MAG. CHS stands for putative naringenin-chalcone synthase. Genes colored black did not have significant matches to the UniRef100 database or were of uncharacterized function. Contigs were blasted using an e-value threshold of 1E-5 and then visualized using Easyfig 2.2.2 [39]. b) Phylogenetic tree of RuBisCO large subunit amino acid sequences from this study (in red) and reference sequences from NCBI nr database and RuBisCO sequences identified as IA subgroup by Bay et al [40,41]. c) Phylogeny of Atacama RuBisCO sequences from this study and
Negev desert RuBisCO sequences from Bay et al 2021 and RuBisCO IE subgroup previously identified from hydrogenotrophic bacteria in Antarctic soil. Node IDs that begin with "Ga" are unbinned sequences identified from the Robison Ridge metagenome [42]. d) Phylogeny of IE subclade from
the original paper by Ji et al. Modified by coloring the ranges for easier comparison with c). Types are designated using previous studies [40–44] as reference. Alignments were made using MUSCLE [37] followed by BMGE v1.12 trimming [38] with default conditions, and the tree was calculated using IQ-TREE v2.1.2 [32] with flags -m MFP -alrt 1000 -bb 1000. Visualization was done using iToL [33] and branches marked with black circles are considered strongly supported (SH-alrt test value > 80, bootstrap value > 95). The full RuBisCO tree file in b) is provided in **Additional File 4**.

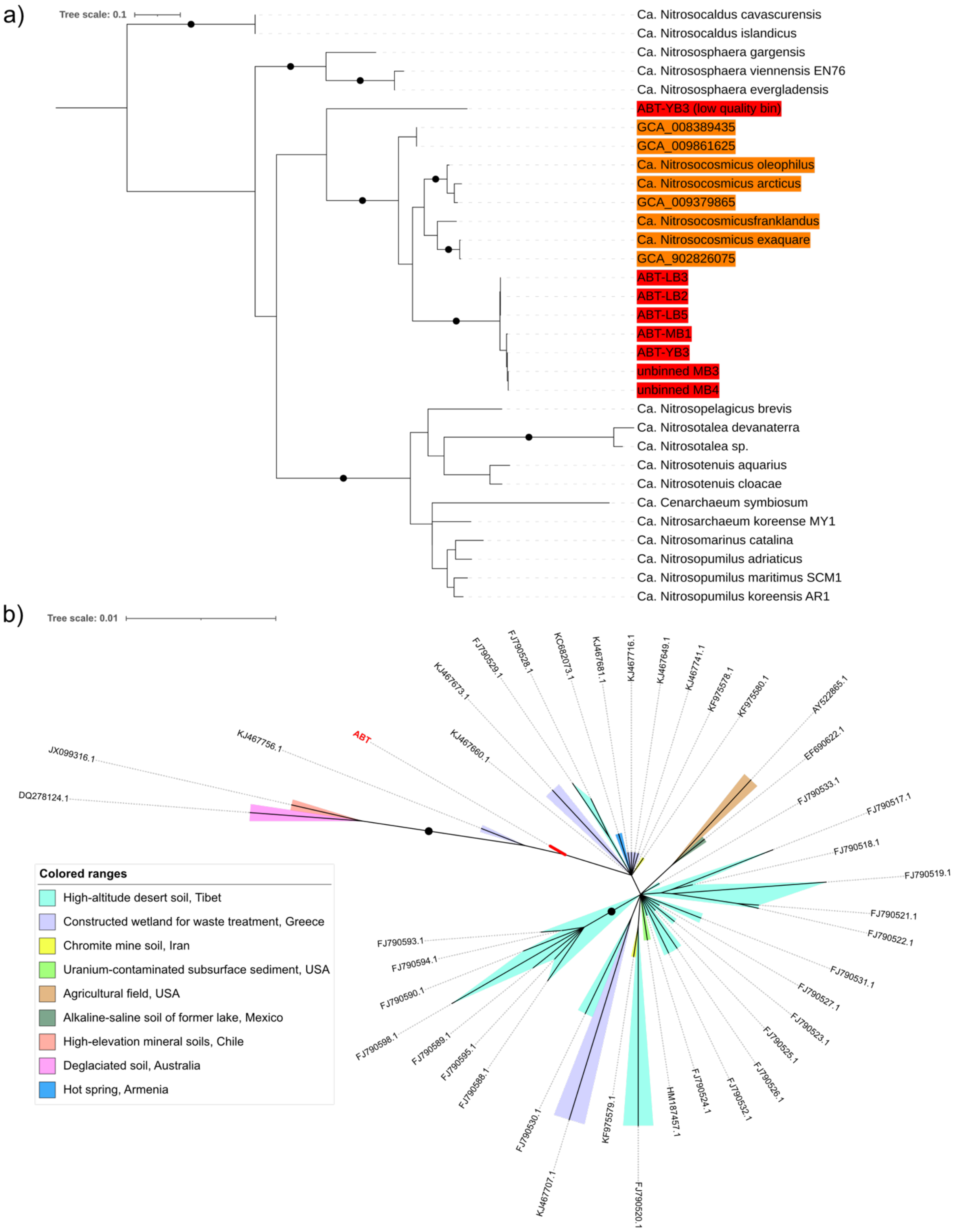
a)
Tree scale: 0.1
Ca. Nitrosocaldus cavascurensis
Ca. Nitrosocaldus islandicus
Ca. Nitrososphaera gargensis
Ca. Nitrososphaera viennensis EN76
Ca. Nitrososphaera evergladensis
ABT-YB3 (low quality bin)
GCA_008389435
GCA_009861625
Ca. Nitrosocosmicus oleophilus
Ca. Nitrosocosmicus arcticus
GCA_009379865
Ca. Nitrosocosmicusfranklandus
Ca. Nitrosocosmicus exaquare
GCA_902826075
ABT-LB3
ABT-LB2
ABT-LB5
ABT-MB1
ABT-YB3
unbinned MB3
unbinned MB4
Ca. Nitrosopelagicus brevis
Ca. Nitrosotalea devanaterra
Ca. Nitrosotalea sp.
Ca. Nitrosotenuis aquarius
Ca. Nitrosotenuis cloacae
Ca. Cenarchaeum symbiosum
Ca. Nitrosarchaeum koreense MY1
Ca. Nitrosomarinus catalina
Ca. Nitrosopumilus adriaticus
Ca. Nitrosopumilus maritimus SCM1
Ca. Nitrosopumilus koreensis AR1
b)
Tree scale: 0.01
Colored ranges
High-altitude desert soil, Tibet
Constructed wetland for waste treatment, Greece
Chromite mine soil, Iran
Uranium-contaminated subsurface sediment, USA
Agricultural field, USA
Alkaline-saline soil of former lake, Mexico
High-elevation mineral soils, Chile
Deglaciated soil, Australia
Hot spring, Armenia
ABT
DQ278124.1
JX099316.1
KJ467756.1
KJ467660.1
KJ467673.1
FJ790529.1
FJ790528.1
KC682073.1
KJ467681.1
KJ467716.1
KJ467649.1
KJ467741.1
KF975578.1
KF975580.1
AY522865.1
EF690622.1
FJ790533.1
FJ790517.1
FJ790518.1
FJ790519.1
FJ790521.1
FJ790522.1
FJ790531.1
FJ790527.1
FJ790523.1
FJ790525.1
FJ790526.1
FJ790532.1
FJ790524.1
HM187457.1
FJ790520.1
KF975579.1
KJ467707.1
FJ790530.1
FJ790588.1
FJ790595.1
FJ790589.1
FJ790598.1
FJ790590.1
FJ790594.1
FJ790593.1

Figure S15. Phylogenies of thaumarchaeal *amoA* and 16S rRNA. a) Tree of all *amoA* nucleotide sequences from this study (red), *amoA* sequences of *Ca.* Nitrosocosmicus (orange) and other representative *Thaumarchaeota* (including *Ca.* Nitrosocaldus used to root the tree). b) Tree of 41 16S rRNA gene sequences that were >99% identical to the recovered 16S rRNA gene sequence from ABT genomes. Node labels correspond to the accession ID and the colored ranges denote the location where the sequence was detected. Alignments were made using MUSCLE [37] followed by BMGE v1.12 trimming [38] with default conditions, and trees were calculated using IQ-TREE v1.5.5 [32] with flags -m MFP -alrt 1000 -bb 1000. Visualization was done using iToL [33] and branches marked with black circles are considered strongly supported (SH-alrt test value > 80, bootstrap value > 95). An additional 16S rRNA gene tree was also made using the SILVA reference NR database sequences that were classified to be in order Nitrosospheraceae and the tree file is provided in **Additional File 4**.

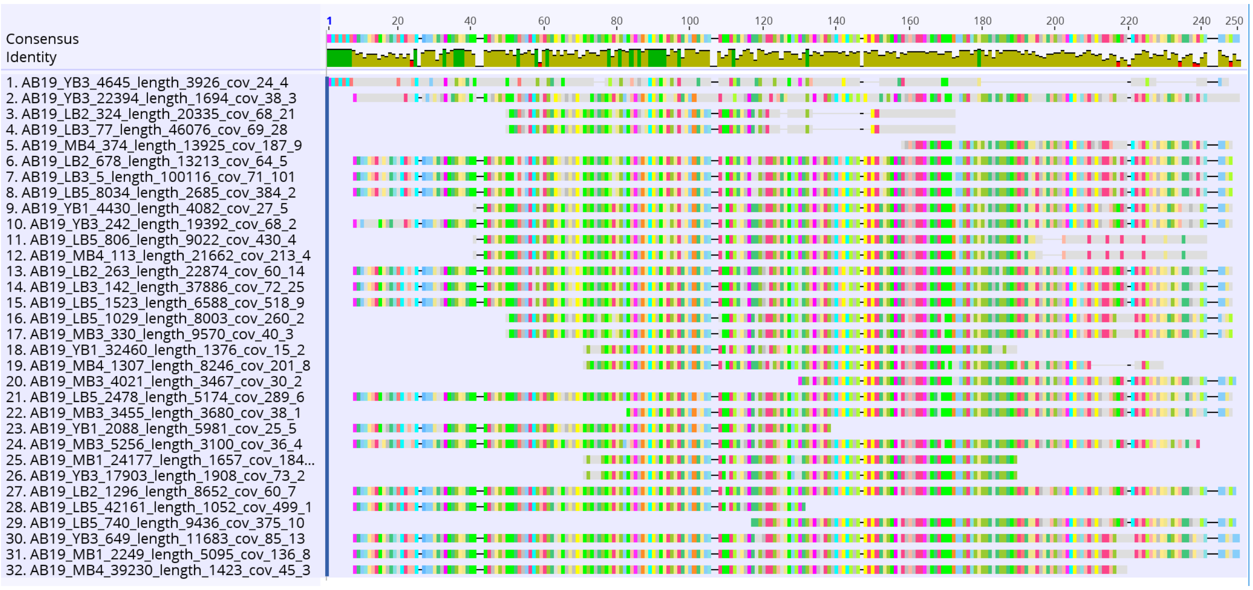

Figure S16. Alignment of 32 aquaporins recovered across all samples. Visualization using the Geneious software. Sequences 1-4 show a high level of sequence divergence, while the rest show truncation at both ends despite most being located mid-scaffold.

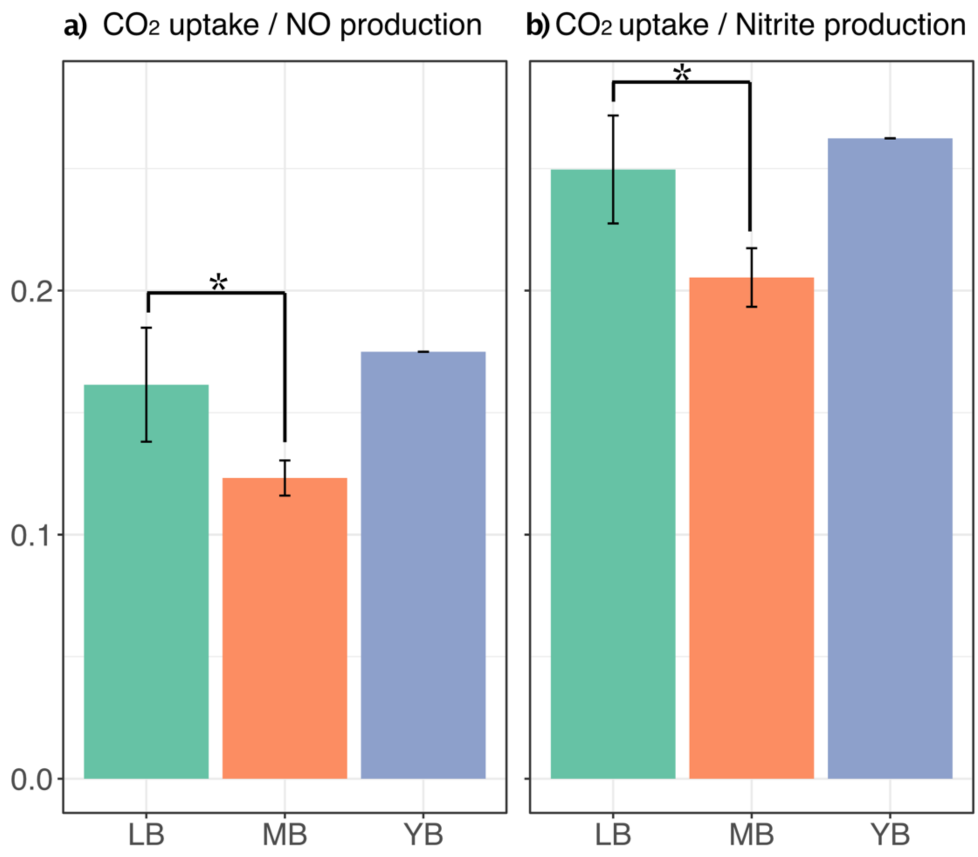

Figure S17. Carbon and nitrogen cycling potentials represented by the genome-scale modeling of *Thaumarchaeota* MAGs in different sampling sites. Individual panels represent the ratio of metabolic fluxes between CO_2 fixation and maximum N output by optimizing the production of NO (a), or nitrite (b). Barcharts represent the average ratios, and error bars represent the standard deviations represented by the modeling of MAGs from each sampling site (i.e. LB, MB and YB). Results from the optimization of N_2O production were not included in the visualization because it produced extremely low C to N flux ratios (<10E-4) from the N_2O maximization. Statistically significant differentiations based on sampling site are identified with an asterisk (Krukal-Wallis test, p-value < 0.05)

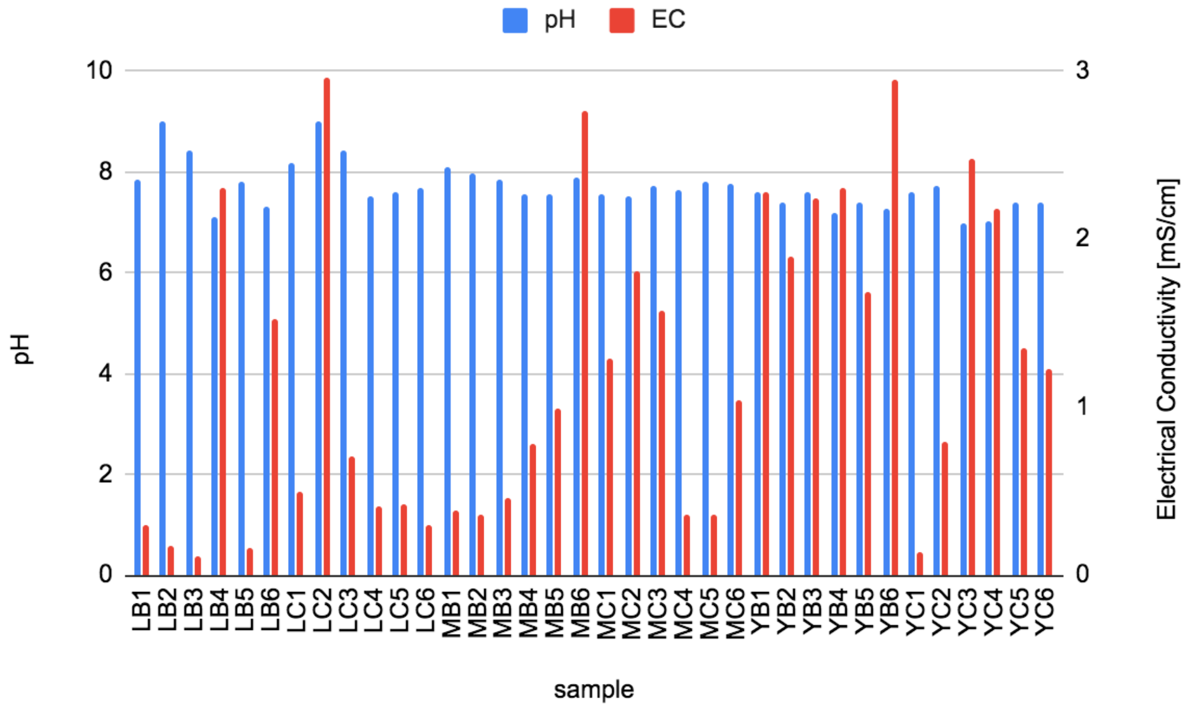

Figure S18. pH and electrical conductivity (EC) of each sample.

a)

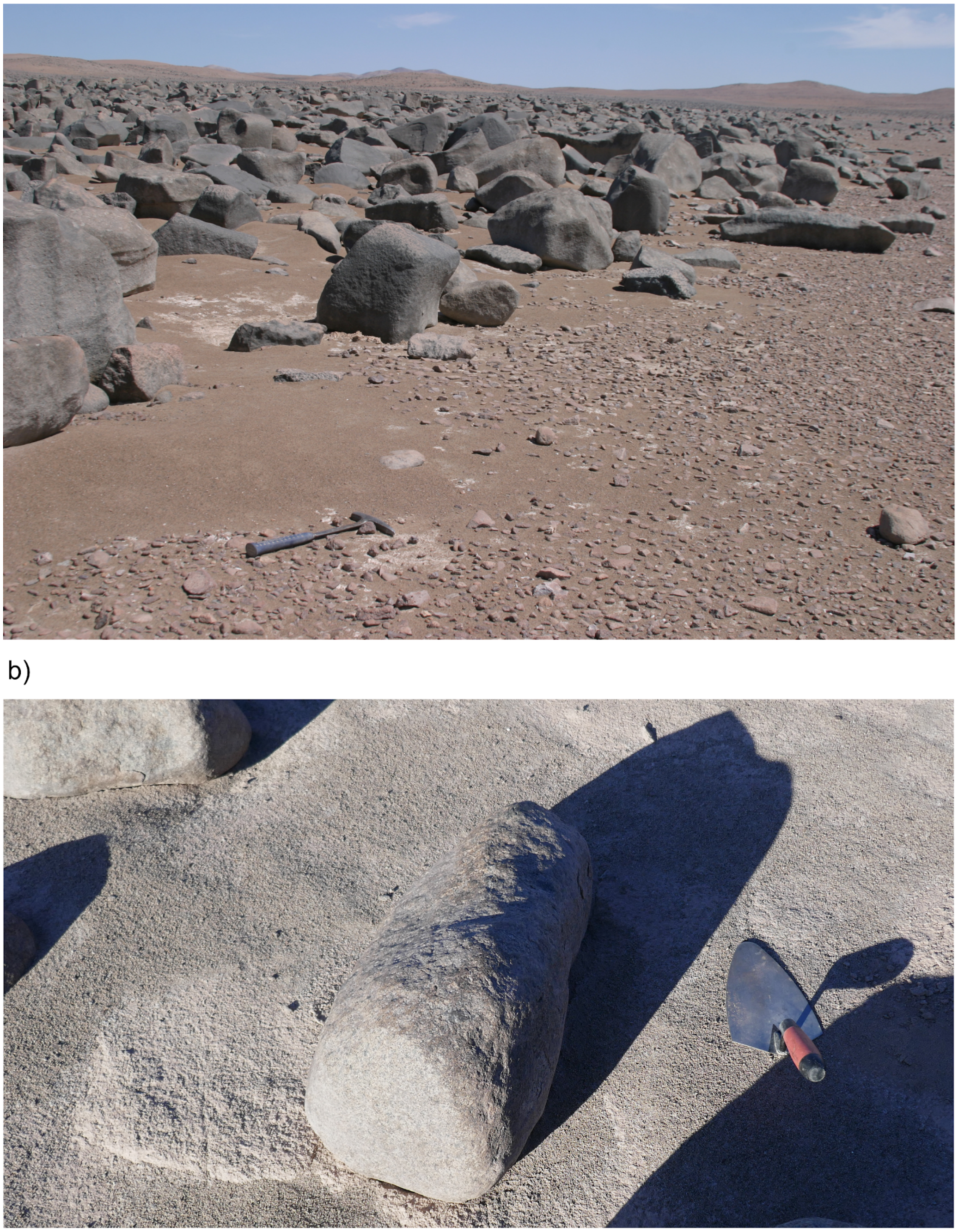

b)

Figure S19. Atacama Boulder Fields. a) A field photo of a densely packed boulder field in the Yungay Valley boulder field. b) A field photo showing a typical boulder chosen for sampling.

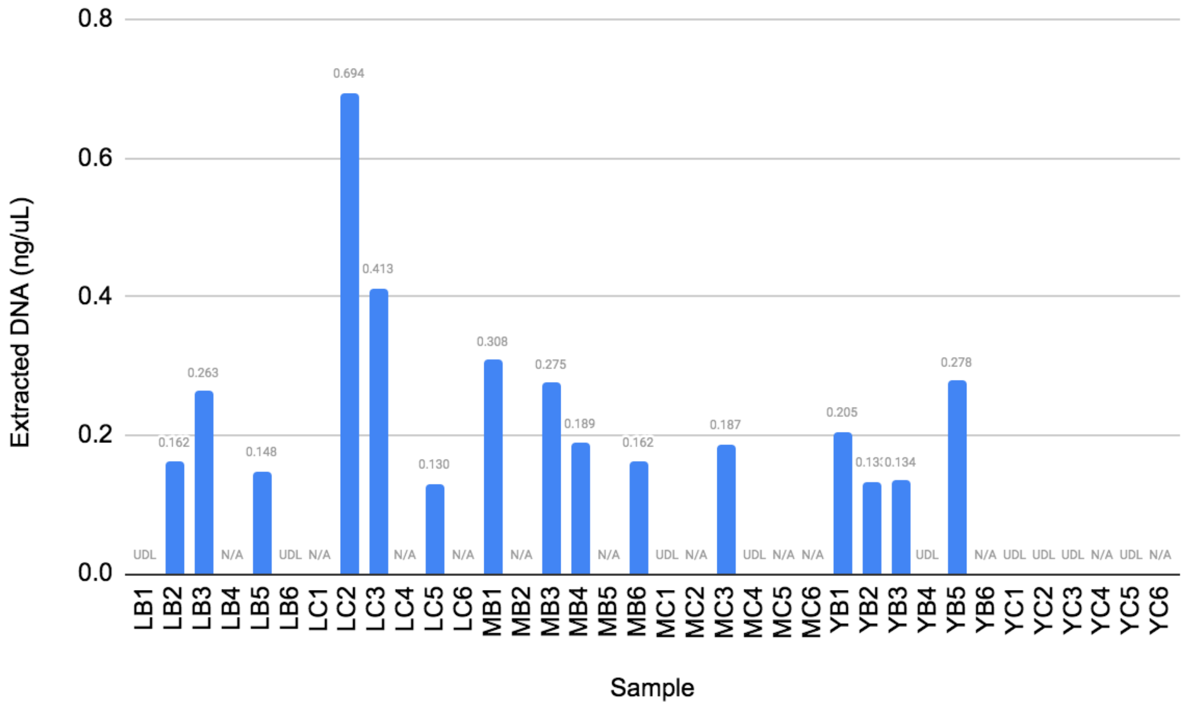

Figure S20. DNA concentrations of metagenomic samples. UDL (under detection limit) indicates extraction was attempted but resulted in DNA amount below detection limit (0.01 ng/uL) and N/A indicates no extraction was attempted.

Tables S3 - S14 are available as a separate Excel file titled Supplementary_Tables:

Table S3. Genome statistics of mid-to-high quality genomes. Mid-to-high quality genomes were determined using CheckM completeness > 75 % and contamination < 10 %. CheckM [44] output (Completeness, contamination, GC std, # ambiguous bases, Genome size, Longest Contig, N50 (scaffolds), Mean scaffold length, # contigs, # scaffolds, # predicted genes, Longest scaffold, GC, N50 (contigs), Coding density, Mean contig length) are accompanied by iRep [45] and GRiD [46] values, rpS3 taxa based on BLAST [35] results against UniRef100 database [36], gtdb-tk classification [31] using "classify_wf", genome coverages and NCBI sample IDs and genome accession numbers.

Table S4. Normalized abundances of all *rpS3* taxa across all samples. Column 1 corresponds to the scaffold containing the centroid of each rpS3 cluster, which was then used to calculate the normalized abundance of each *rpS3* cluster (taxa) across all samples.

Table S5. Locus information and blast output of putative genes for ammonification.

Table S6. Statistics and meta-data of the reference genomes used for comparative genomics.

Table S7. Orthologous protein clusters with GO [47,48] **and Swiss-Prot** [49] **annotation.** Clusters were determined using Orthovenn2 [50]. In orange are clusters with putative functions associated with stress response and in yellow are clusters with putative functions associated with nitrogen metabolism.

Table S8. List of *amoABCX* genes, 4HB/3HP pathway genes, TCA cycle, gluconeogenesis, pentose phosphate pathway and other notable genes for each ABT genomes.

Table S9. Singletons of LB3, MB4, YB3 genomes. Singleton genes for which no orthologs were found in any of the six genomes analyzed using Orthovenn2 [50]. Genes were annotated by searching [35,50] against UniRef100 database [36]

Table S10. Ion chromatography raw data in mg/g soil.

Table S11. List of NCBI genomes used for phylogenomic tree construction. NCBI genomes classified as Thaumarchaeota on 30th May 2020, filtered using CheckM completeness >50 % and contamination < 5%.

Additional Files:

Additional File 1: Newick treefile of *Ca. Nitrosodeserticola* and NCBI genomes annotated as *Thaumarchaeaota.*

Additional File 2: *Thaumarchaeota* genome-scale metabolic model parameters and simulation conditions.

Additional File 3: Newick treefile of all RuBisCO sequences found in this study and those extracted from NCBI reference genomes and reported in Bay et al [40].

Additional File 4: Newick treefile of Ca. *Nitrosodeserticola* 16S rRNA gene sequences and SILVA 138.1 Reference NR database sequences (accessed 06/08/2021) that were classified to be in order *Nitrososphaeraceae*. Alignment was made using MUSCLE [37] followed by BMGE v1.12 trimming [38] with default conditions, and the tree was calculated using IQ-TREE v2.1.2 [32] with flags -m MFP -alrt 1000 -bb 1000.

Supplementary references

1. Lawrence MG. The Relationship between Relative Humidity and the Dewpoint Temperature in Moist Air: A Simple Conversion and Applications. Bull Am Meteorol Soc. American Meteorological Society; 2005;86:225–34.

2. Oksanen J, Blanchet FG, Kindt R, Legendre P, Minchin P. The vegan package: Community Ecology Package. R package version 2.0--2. 2011;

3. Core Team R, Others. R: A language and environment for statistical computing. Vienna, Austria: R Foundation for Statistical Computing. Available. 2013;

4. Wentworth CK. A Scale of Grade and Class Terms for Clastic Sediments. J Geol. The University of Chicago Press; 1922;30:377–92.

5. Udden JA. Mechanical composition of clastic sediments. GSA Bulletin. GeoScienceWorld; 1914;25:655–744.

6. Sager C, Airo A, Arens FL, Rabethge C, Schulze-Makuch D. New types of boulder accumulations in the hyper-arid Atacama Desert. Geomorphology . 2020;350:106897.

7. Team QD, Others. QGIS geographic information system. Open source geospatial foundation project. 2016;

8. Kramm, Kramm, Hoffmeister. A Relief Dependent Evaluation of Digital Elevation Models on Different Scales for Northern Chile [Internet]. ISPRS International Journal of Geo-Information. 2019. p. 430. Available from: http://dx.doi.org/10.3390/ijgi8100430

9. Schneider CA, Rasband WS, Eliceiri KW. NIH Image to ImageJ: 25 years of image analysis. Nat Methods. 2012;9:671–5.

10. Zhang Y, Sievert SM. Pan-genome analyses identify lineage- and niche-specific markers of evolution and adaptation in Epsilonproteobacteria. Front Microbiol. 2014;5:110.

11. Fu L, Niu B, Zhu Z, Wu S, Li W. CD-HIT: accelerated for clustering the next-generation sequencing data. Bioinformatics. 2012;28:3150–2.

12. Li F, Xie W, Yuan Q, Luo H, Li P, Chen T, et al. Genome-scale metabolic model analysis indicates low energy production efficiency in marine ammonia-oxidizing archaea. AMB Express. 2018;8:106.

13. Pimentel ZT, Dufault-Thompson K, Russo KT, Scro AK, Smolowitz RM, Gomez-Chiarri M, et al. Microbiome Analysis Reveals Diversity and Function of Mollicutes Associated with the Eastern Oyster, Crassostrea virginica. mSphere [Internet]. 2021;6. Available from: http://dx.doi.org/10.1128/mSphere.00227-21

14. He C, Keren R, Whittaker ML, Farag IF, Doudna JA, Cate JHD, et al. Genome-resolved metagenomics reveals site-specific diversity of episymbiotic CPR bacteria and DPANN archaea in groundwater ecosystems. Nat Microbiol. 2021;6:354–65.

15. Khachikyan A, Milucka J, Littmann S, Ahmerkamp S, Meador T, Könneke M, et al. Direct Cell Mass Measurements Expand the Role of Small Microorganisms in Nature. Appl Environ Microbiol [Internet]. 2019;85. Available from: http://dx.doi.org/10.1128/AEM.00493-19

16. Dufault-Thompson K, Steffensen JL, Zhang Y. Using PSAMM for the Curation and Analysis of Genome-Scale Metabolic Models. Methods Mol Biol. 2018;1716:131–50.

17. Steffensen JL, Dufault-Thompson K, Zhang Y. PSAMM: A Portable System for the Analysis of Metabolic Models. PLoS Comput Biol. 2016;12:e1004732.

18. Kramm, Kramm, Hoffmeister. A Relief Dependent Evaluation of Digital Elevation Models on Different Scales for Northern Chile [Internet]. ISPRS International Journal of Geo-Information. 2019. p. 430. Available from: http://dx.doi.org/10.3390/ijgi8100430

19. Matmon A, Quade J, Placzek C, Fink D, Arnold M, Aumaître G, et al. Seismic origin of the Atacama Desert boulder fields. Geomorphology . 2015;231:28–39.

20. Quade J, Reiners P, Placzek C, Matmon A, Pepper M, Ojha L, et al. Seismicity and the strange rubbing boulders of the Atacama Desert, northern Chile. Geology. GeoScienceWorld; 2012;40:851–4.

21. Reid JB, Bucklin EP, Copenagle L, Kidder J, Pack SM, Polissar PJ, et al. Sliding rocks at the Racetrack, Death Valley: What makes them move? Geology. GeoScienceWorld; 1995;23:819–22.

22. Shelton JS. Can Wind Move Rocks on Racetrack Playa? Science. 1953;117:438–9.

23. Dorn R, Krinsley D. NEW PERSPECTIVES ON COLLUVIAL BOULDER DEPOSITS IN THE SOUTHWESTERN GREAT BASIN, USA. Phys Geogr. Taylor & Francis; 1994;15:62–79.

24. Cluer JK. Leveed boulder flows on volcanic slopes of the Sonoran Desert, Arizona. J Arid Environ. Academic Press; 1988;15:43–52.

25. Friend DA, Phillips FM, Campbell SW, Liu T, Sharma P. Evolution of desert colluvial boulder slopes. Geomorphology . 2000;36:19–45.

26. Burstein D, Sun CL, Brown CT, Sharon I, Anantharaman K, Probst AJ, et al. Major bacterial lineages are essentially devoid of CRISPR-Cas viral defence systems. Nat Commun. 2016;7:10613.

27. Ledbetter RN, Garcia Costas AM, Lubner CE, Mulder DW, Tokmina-Lukaszewska M, Artz JH, et al. The Electron Bifurcating FixABCX Protein Complex from Azotobacter vinelandii: Generation of Low-Potential Reducing Equivalents for Nitrogenase Catalysis. Biochemistry. 2017;56:4177–90.

28. Reji L, Francis CA. Metagenome-assembled genomes reveal unique metabolic adaptations of a basal marine Thaumarchaeota lineage. ISME J [Internet]. 2020; Available from: http://dx.doi.org/10.1038/s41396-020-0675-6

29. Nicol GW, Hink L, Gubry-Rangin C, Prosser JI, Lehtovirta-Morley LE. Genome Sequence of “ Nitrosocosmicus franklandus” C13, a Terrestrial Ammonia-Oxidizing Archaeon. Microbiol Resour Announc [Internet]. 2019;8. Available from: http://dx.doi.org/10.1128/MRA.00435-19

30. Houston J, Hartley AJ. The central Andean west-slope rainshadow and its potential contribution to the origin of hyper-aridity in the Atacama Desert [Internet]. International Journal of Climatology. 2003. p. 1453–64. Available from: http://dx.doi.org/10.1002/joc.938

31. Chaumeil P-A, Mussig AJ, Hugenholtz P, Parks DH. GTDB-Tk: a toolkit to classify genomes with the Genome Taxonomy Database. Bioinformatics [Internet]. 2019; Available from: http://dx.doi.org/10.1093/bioinformatics/btz848

32. Nguyen L-T, Schmidt HA, von Haeseler A, Minh BQ. IQ-TREE: a fast and effective stochastic algorithm for estimating maximum-likelihood phylogenies. Mol Biol Evol. 2015;32:268–74.

33. Letunic I, Bork P. Interactive tree of life (iTOL) v3: an online tool for the display and annotation of phylogenetic and other trees. Nucleic Acids Res. 2016;44:W242–5.

34. Wickham H, Chang W. ggplot2: an implementation of the grammar of graphics.(0.9. 3 edn). See http://ggplot2 org. 2012;

35. Altschul SF, Gish W, Miller W, Myers EW, Lipman DJ. Basic local alignment search tool. J Mol Biol. 1990;215:403–10.

36. Suzek BE, Huang H, McGarvey P, Mazumder R, Wu CH. UniRef: comprehensive and non-redundant UniProt reference clusters. Bioinformatics. 2007;23:1282–8.

37. Edgar RC. MUSCLE: multiple sequence alignment with high accuracy and high throughput. Nucleic Acids Res. 2004;32:1792–7.

38. Criscuolo A, Gribaldo S. BMGE (Block Mapping and Gathering with Entropy): a new software for selection of phylogenetic informative regions from multiple sequence alignments. BMC Evol Biol. 2010;10:210.

39. Sullivan MJ, Petty NK, Beatson SA. Easyfig: a genome comparison visualizer. Bioinformatics. 2011;27:1009–10.

40. Bay SK, Waite DW, Dong X, Gillor O, Chown SL, Hugenholtz P, et al. Chemosynthetic and photosynthetic bacteria contribute differentially to primary production across a steep desert aridity gradient. ISME J. Nature Publishing Group; 2021;1–18.

41. Grostern A, Alvarez-Cohen L. RubisCO-based CO2 fixation and C1 metabolism in the actinobacterium Pseudonocardia dioxanivorans CB1190. Environ Microbiol. 2013;15:3040–53.

42. Park SW, Park ST, Lee JE, Kim YM. Pseudonocardia carboxydivorans sp. nov., a carbon monoxide-oxidizing actinomycete, and an emended description of the genus Pseudonocardia [Internet]. INTERNATIONAL JOURNAL OF SYSTEMATIC AND EVOLUTIONARY MICROBIOLOGY. 2008. p. 2475–8. Available from: http://dx.doi.org/10.1099/ijs.0.65765-0

43. Banda DM, Pereira JH, Liu AK, Orr DJ, Hammel M, He C, et al. Novel bacterial clade reveals origin of form I Rubisco. Nat Plants. 2020;6:1158–66.

44. Parks DH, Imelfort M, Skennerton CT, Hugenholtz P, Tyson GW. CheckM: assessing the quality of microbial genomes recovered from isolates, single cells, and metagenomes. Genome Res. 2015;25:1043–55.

45. Brown CT, Olm MR, Thomas BC, Banfield JF. Measurement of bacterial replication rates in microbial communities [Internet]. Nature Biotechnology. 2016. p. 1256–63. Available from: http://dx.doi.org/10.1038/nbt.3704

46. Emiola A, Oh J. High throughput in situ metagenomic measurement of bacterial replication at ultra-low sequencing coverage. Nat Commun. 2018;9:4956.

47. Ashburner M, Ball CA, Blake JA, Botstein D, Butler H, Michael Cherry J, et al. Gene Ontology: tool for the unification of biology [Internet]. Nature Genetics. 2000. p. 25–9. Available from: http://dx.doi.org/10.1038/75556

48. The Gene Ontology Consortium. The Gene Ontology Resource: 20 years and still GOing strong. Nucleic Acids Res. 2019;47:D330–8.

49. Bairoch A, Apweiler R. The SWISS-PROT protein sequence database and its supplement TrEMBL in 2000. Nucleic Acids Res. 2000;28:45–8.

50. Xu L, Dong Z, Fang L, Luo Y, Wei Z, Guo H, et al. OrthoVenn2: a web server for whole-genome comparison and annotation of orthologous clusters across multiple species. Nucleic Acids Res. 2019;47:W52–8.
